# Metabolic Effect of Blocking Sodium-Taurocholate Co-Transporting Polypeptide in Hypercholesterolemic Humans with a Twelve-Week Course of Bulevirtide—An Exploratory Phase I Clinical Trial

**DOI:** 10.3390/ijms232415924

**Published:** 2022-12-14

**Authors:** Felicitas Stoll, Andrea Seidel-Glätzer, Ina Burghaus, Oliver Göring, Max Sauter, Peter Rose, Volker Daniel, Mathias Haag, Matthias Schwab, Johannes Riffel, Florian André, Lenka Taylor, Johanna Weiss, Jürgen Burhenne, Volker Cleeves, Walter E. Haefeli, Antje Blank

**Affiliations:** 1 Department of Clinical Pharmacology and Pharmacoepidemiology, Heidelberg University Hospital, 69120 Heidelberg, Germany; 2 Coordination Centre for Clinical Trials (KKS) Heidelberg, 69120 Heidelberg, Germany; 3 Institute of Immunology, Heidelberg University Hospital, 69120 Heidelberg, Germany; 4 Dr. Margarete Fischer-Bosch-Institute of Clinical Pharmacology, 70376 Stuttgart, Germany; 5 University of Tuebingen, 72076 Tuebingen, Germany; 6 Departments of Clinical Pharmacology, and of Biochemistry and Pharmacy, University of Tuebingen, 72076 Tuebingen, Germany; 7 Cluster of Excellence iFIT (EXC2180) “Image-Guided and Functionally Instructed Tumor Therapies”, University of Tübingen, 72076 Tübingen, Germany; 8 Department of Cardiology, Angiology and Pneumology, Heidelberg University Hospital, 69120 Heidelberg, Germany; 9 Pharmacy, Heidelberg University Hospital, 69120 Heidelberg, Germany; 10 Technology Transfer Heidelberg GmbH, 69120 Heidelberg, Germany

**Keywords:** LDL cholesterol, bulevirtide, bile acid, NTCP, phase I clinical trial

## Abstract

Bile acids (BA) play an important role in cholesterol metabolism and possess further beneficial metabolic effects as signalling molecules. Blocking the hepatocellular uptake of BA via sodium-taurocholate co-transporting polypeptide (NTCP) with the first-in-class drug bulevirtide, we expected to observe a decrease in plasma LDL cholesterol. In this exploratory phase I clinical trial, volunteers with LDL cholesterol > 130 mg/dL but without overt atherosclerotic disease were included. Thirteen participants received bulevirtide 5 mg/d subcutaneously for 12 weeks. The primary aim was to estimate the change in LDL cholesterol after 12 weeks. Secondary endpoints included changes in total cholesterol, HDL cholesterol, lipoprotein(a), inflammatory biomarkers, and glucose after 12 weeks. In addition, cardiac magnetic resonance imaging (CMR) was performed at four time points. BA were measured as biomarkers of the inhibition of hepatocellular uptake. After 12 weeks, LDL cholesterol decreased not statistically significantly by 19.6 mg/dL [−41.8; 2.85] (Hodges–Lehmann estimator with 95% confidence interval). HDL cholesterol showed a significant increase by 5.5 mg/dL [1.00; 10.50]. Lipoprotein(a) decreased by 1.87 mg/dL [−7.65; 0]. Inflammatory biomarkers, glucose, and cardiac function were unchanged. Pre-dose total BA increased nearly five-fold (from 2026 nmol/L ± 2158 (mean ± SD) at baseline to 9922 nmol/L ± 7357 after 12 weeks of treatment). Bulevirtide was generally well tolerated, with most adverse events being administration site reactions. The exploratory nature of the trial with a limited number of participants allows the estimation of potential effects, which are crucial for future pharmacological research on bile acid metabolism in humans.

## 1. Introduction

Atherosclerotic cardiovascular (CV) disease remains the leading cause of death and a major burden of morbidity. The increasing prevalence of obesity and diabetes mellitus poses a pivotal challenge in the future prevention of CV disease [1]. Innovative pharmaceutical strategies are urgently needed to meet this challenge.

In preclinical research, there is a profound interest in the metabolic roles of bile acids (BA) as proposed innovative pharmacological targets. This has two reasons: first, BA metabolism is directly linked to cholesterol turnover [2,3]. Second, BA play an important role as signalling molecules in metabolic regulation [4,5,6]. These two properties make BA regulation a promising pharmacological target for primary and secondary CV prevention. This understanding is supported by observations of altered BA plasma concentrations and excretion in conditions related to CV risk: BA concentration in peripheral blood are elevated in obesity and diabetes type II [4]. At the same time, postoperative increases in BA levels have been associated with the remission of diabetes mellitus II after Roux-en-Y gastric bypass [7].

BA are synthesised from cholesterol. The rate-limiting enzyme of BA synthesis is CYP7A1; alternative pathways make up only about 6% [5]. Induction of CYP7A1 leads to the consumption of hepatic cholesterol, resulting in the upregulation of LDL receptor expression and, ultimately, in a decrease in plasma LDL cholesterol [5]. Loss of CYP7A1 function leads to statin-resistant hypercholesteremia [8]. CYP7A1 expression is suppressed by activation of the transcriptional repressor small heterodimer partner 1 (SHP) via the nuclear farnesoid X receptor (FXR). Activating ligands of FXR, BA regulate gene expression and thus BA synthesis from cholesterol in the hepatocytes [2,5]. FXR activation by obeticholic acid (a synthetic BA derivative) in patients with steatohepatitis led to a significant increase in total and LDL cholesterol and a decrease in HDL cholesterol within the first 12 weeks of treatment [9]. Hence, inhibition of FXR activation likely reverses this effect.

Subject to enterohepatic circulation, BA are actively and to a large part (about 95%) reabsorbed in the terminal ileum after being secreted into the bile [5]. From the portal vein, they are transported into the hepatocyte on the basolateral side by the sodium-taurocholate co-transporting polypeptide (NTCP/SLC10A1) and by the (sodium-independent) organic anion transporting polypeptides (OATP). NTCP is responsible for 80% of BA uptake [5]. BA uptake is downregulated by FXR [10].

Bulevirtide (Hepcludex^®^; pre-authorisation name: myrcludex B) is a first-in-class medicine that recently received market authorization to treat patients with hepatitis B and D co-infection. The N-terminal myristoylated and C-terminal amidated 47-amino acid lipopeptide, which was derived from the large S antigen of the hepatitis B virus, was found to block entry of hepatitis B virus into hepatocytes by highly effective inhibition of NTCP [11,12,13,14,15,16].

Mediated by the interaction of the hepatitis B virus with NTCP, humanized infected mice showed an induction of CYP7A1, bringing forth compensatory BA production from cholesterol. This effect could be mimicked by the administration of bulevirtide to uninfected mice [17,18]. Because of NTCP blockage, hepatic BA clearance is partially inhibited, and plasma BA increases profoundly. By prolonged BA signalling, NTCP inhibition with bulevirtide in obese OATP1a/1b KO mice led to elevated faecal energy output and bodyweight reduction and induced GLP-1 secretion [19].

Based on evidence from these mouse models, we hypothesized that NTCP blockage by bulevirtide could be successfully used to decrease LDL cholesterol in humans, while we expected the increased plasma level of BA to translate into further favourable effects on plasma glucose and endothelial function. We present the results of an exploratory, pilot phase I trial investigating the effect of bulevirtide on lipid metabolism, glucose homeostasis, inflammatory biomarkers, and myocardial tissue and function in hypercholesterolemic volunteers.

## 2. Results

### 2.1. Baseline Characteristics

Fifty-seven volunteers were screened, and fourteen volunteers were enrolled in the trial, and their baseline characteristics are given in Table 1 with a focus on their cardiovascular risk profile. Only 14 of the planned 20 volunteers were enrolled because recruitment was difficult; it was stopped prematurely in the face of expiring public funding. The main reasons for screening failure were too low total and/or LDL cholesterol and relevant abnormalities in medical assessment or relevant medical disorders. Thirteen participants (=per-protocol set) received at least 80% of the study medication for at least 10 weeks and showed a proportion of days covered of 99.8%. One participant dropped out because of an SAE.

### 2.2. Primary Analysis

**Figure 1:**
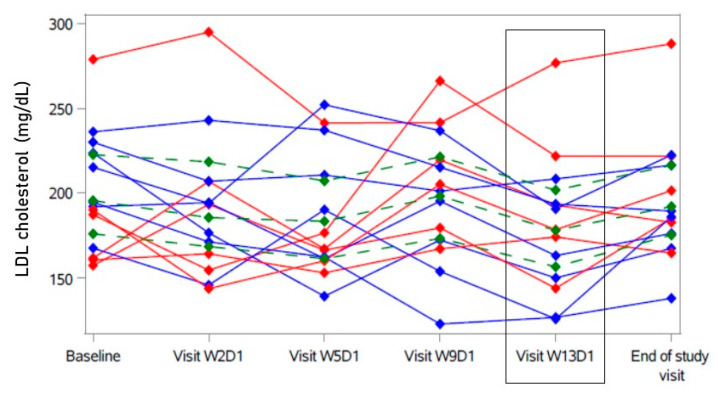
Course of LDL cholesterol under bulevirtide treatment in individual participants. Primary endpoint was change in LDL cholesterol after 12 weeks of treatment (Visit W13D1, highlighted): Participants with decrease of LDL cholesterol > 19.6 mg/dL in blue (“responder”); participants with no decrease and decrease < 19.6 mg/dL of LDL cholesterol in red (“non-responder”). Green: Hodges–Lehmann estimator with 95% confidence interval.

After 12 weeks (W13D1), LDL cholesterol had decreased by 19.6 mg/dL [−41.80; 2.85] (Hodges–Lehmann estimator with 95% confidence interval, see Table 2 and Figure 1). Sensitivity analyses showed similar results. This change was not statistically significant compared to the baseline (see Table 2). At the individual level, seven participants showed a decrease in LDL cholesterol of more than 19.6 mg/dL (“responder” in blue, Figure 1), while in six participants, a decrease of less than 19.6 mg/dL or no decrease was observed (“non-responder” in red, Figure 1). With the exception of one outlier, “non-responders” tended to have a comparably lower baseline LDL cholesterol (≤190 mg/dL) (Figure 1). Visually, a rise in LDL cholesterol after eight weeks (W9D1) compared to the previous visit (no increase compared to baseline) was apparent that was mostly driven by the “non-responders” (Figure 1 and Supplementary Materials). Most “responders” showed an increase in LDL cholesterol after the treatment of bulevirtide had been ended for one month (end-of-study visit) (Figure 1 and Supplementary Materials).

### 2.3. Secondary Analyses

**Figure 2:**
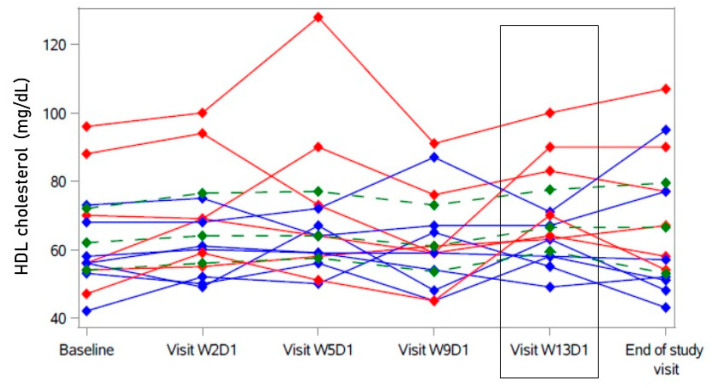
Course of HDL cholesterol under bulevirtide treatment in individual participants. A secondary endpoint was change in HDL cholesterol after 12 weeks of treatment (Visit W13D1, highlighted). Participants with decrease of LDL cholesterol (primary endpoint) > 19.6 mg/dL in blue (“responder”); participants with no decrease and decrease < 19.6 mg/dL of LDL cholesterol in red (“non-responder”). Green: Hodges–Lehmann estimator with 95% confidence interval.

HDL cholesterol was statistically significantly higher after 12 weeks (W13D1) than at baseline (see Table 2 and Supplementary Materials). However, this change did not show consistency over the twelve-week course of treatment (see Figure 2 and Supplementary Materials). The relative increase in LDL cholesterol after 8 weeks (W9D1, Figure 1) in some participants was accompanied by a temporary decrease in HDL cholesterol (−0.5 mg/dL [−6.5; 7.5] compared to baseline in all, see Supplementary Materials), also mostly driven by the “non-responders” (red in Figure 2).

There was no statistically significant change in total cholesterol, triglycerides, and apolipoprotein B. During treatment, lipoprotein(a) values were consistently but not statistically significantly lower than at baseline (see Table 2 and Supplementary Materials).

**Figure 3:**
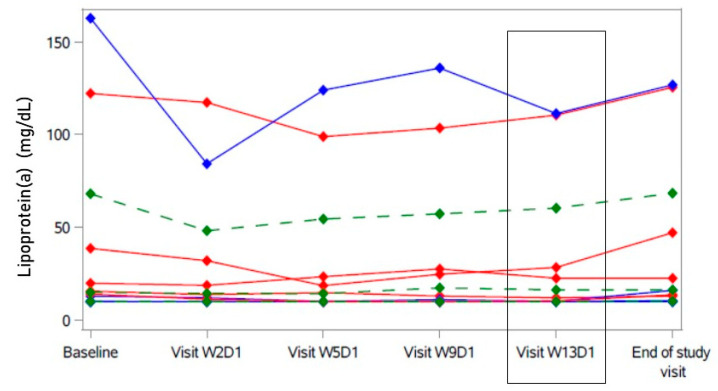
Course of lipoprotein(a) under bulevirtide treatment in individual participants. A secondary endpoint was change in lipoprotein(a) after 12 weeks of treatment (Visit W13D1, highlighted). Participants with decrease of LDL cholesterol (primary endpoint) > 19.6 mg/dL in blue (“responder”); participants with no decrease and decrease < 19.6 mg/dL of LDL cholesterol in red (“non-responder”). Green: Hodges–Lehmann estimator with 95% confidence interval.

Looking at individual data, this decrease appeared to be majorly driven by the participant with the highest baseline lipoprotein(a) level, who also showed a response in LDL cholesterol (Figure 3). The nadir of lipoprotein(a) in this participant was reached after one week of treatment with bulevirtide (W2D1); still, all later values remained below the baseline level. There was no participant with a lipoprotein(a) level > 180 mg/dL, associated with an extremely high lifetime risk of atherosclerotic cardiovascular disease [20].

hs-CRP did not change statistically significantly during the course of treatment. There was no statistically significant change in IL-1b, IL-6, E-selectin, ICAM-1, TGF-β1, and neopterin (see Table 3). After 12 weeks of bulevirtide treatment, TNF-α did not statistically significantly differ from baseline but showed a transient increase at week 9 (see Table 3 and Supplementary Materials).

Cardiac magnetic resonance imaging (CMR) showed normal left ventricular function (left ventricular ejection fraction, LVEF) at baseline and after 12 weeks without any statistically or clinically relevant changes. Strain imaging did not reveal any changes after 12 weeks. Mean global T1 and T2 time did not significantly change either (see Table 3).

Neither HbA1c nor homeostasis model assessment (HOMA) significantly differed after the 12-week treatment course (see Table 3).

After 12 weeks of bulevirtide, total pre-dose BA (trough concentration before administration of bulevirtide) increased 4.9-fold (from 2026 nmol/L ± 2158 to 9922 nmol/L ± 7357) and returned to 1928 nmol/L ± 1666 one month after the end of treatment. The AUC of the conjugated BA generally increased more than the AUC of unconjugated BA, with the most pronounced, 15-fold increase of the AUC of taurocholic acid (TCA), a biomarker for the NTCP-blocking effect of bulevirtide [21]. The detailed BA data are presented in the Supplementary Materials.

**Figure 4:**
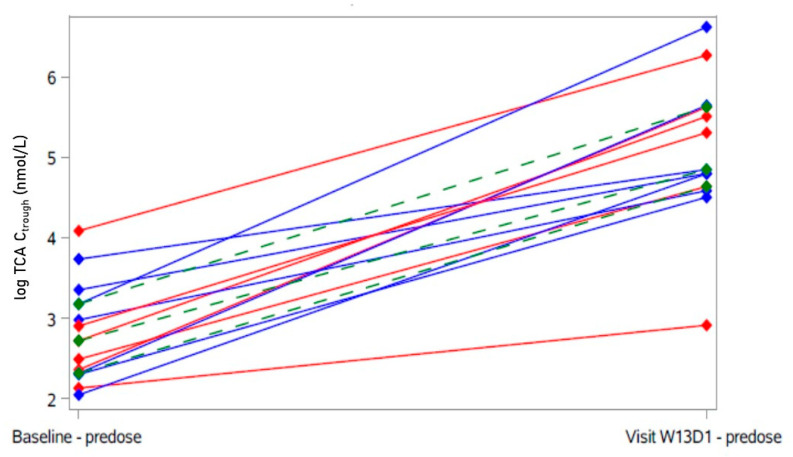
Individual taurocholic acid (TCA) trough concentrations after 12 weeks of treatment with bulevirtide. Participants with decrease of LDL cholesterol > 19.6 mg/dL in blue (“responder”); participants with no decrease and decrease < 19.6 mg/dL of LDL cholesterol in red (“non-responder”). Green: Hodges–Lehmann estimator with 95% confidence interval.

**Figure 5:**
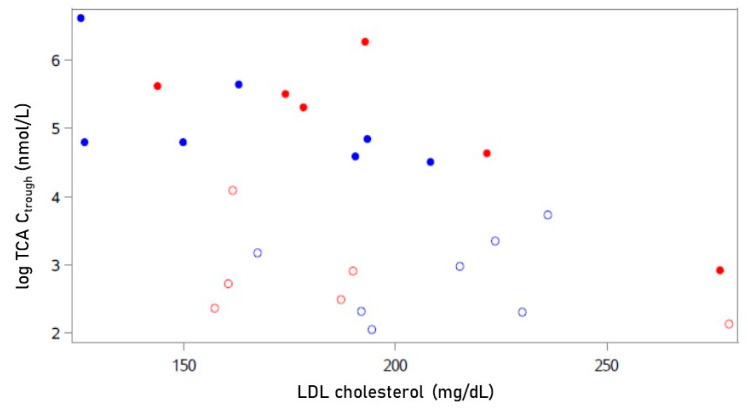
Correlation between taurocholic acid (TCA) trough concentrations and plasma LDL cholesterol at baseline (open circles) and after 12 weeks (closed circles) of treatment with bulevirtide (Spearman r = −0.60; *p* = 0.03 for values after 12 weeks, closed circles). Participants with decrease of LDL cholesterol > 19.6 mg/dL in blue (“responder”); participants with no decrease and decrease < 19.6 mg/dL of LDL cholesterol in red (“non-responder”).

There was a statistically significant negative correlation between the plasma level of LDL cholesterol and TCA trough concentration (= pre-dose bulevirtide, see Figure 4) after 12 weeks of treatment (Spearman r = −0.60, *p* = 0.03, see Figure 5; correlation at baseline: non-significant), indicating an impact of the NTCP-blocking effect of bulevirtide on the plasma level of LDL cholesterol. There was one participant with a comparably small level of TCA. This participant also had the highest baseline level of LDL cholesterol of all and was a “non-responder” to bulevirtide treatment (decrease in LDL cholesterol < 19.6 mg/dL) (see Figure 4 and Figure 5).

The pharmacokinetic parameters of bulevirtide have been published separately [22].

### 2.4. Safety Assessment

Bulevirtide was well tolerated. Sixty-eight adverse events were observed (mild: n = 52, moderate: 13, severe: 3 (blood pressure increased, syncope, lipase increased; all resolved)). Causality with investigational medicinal product (IMP) was assessed as definite in 11 cases, probable in 10 cases, and possible in 9 cases. General disorders and administration site conditions (e.g., injection site haematoma, erythema, pruritus, pallor) were reported by eight participants. Alanine aminotransferase mildly increased in three participants, and white blood cell count mildly decreased in two.

There was one serious adverse event (syncope with subsequent hospitalization and increase of troponin without confirmed myocardial infarction); even though a causality with the IMP was considered unlikely, study treatment was stopped in this participant.

In all 12-lead ECGs normal at baseline (n = 9), no abnormalities were reported during study treatment and at the end-of-study visit. In five ECGs, there were findings at baseline judged as abnormal; however, they did not represent a contraindication for study participation (sinus bradycardia, AV block I°, incomplete RBBB, signs of left atrial hypertrophy). In one participant, monomorphic ventricular extrasystoles were observed on ECG after four weeks of treatment; ECGs at the following visits did not show any clinically significant changes. Throughout the study, we observed no new conduction delays. Average heart rate and conduction times at screening and after 12 weeks of treatment are presented in the Supplementary Materials.

## 3. Discussion

An ideal antiatherosclerosis treatment would combine LDL cholesterol lowering with further beneficial metabolic effects, e.g., an improvement of glucose homeostasis and loss of excess weight. We investigated the effect of elevated BA, which gained interest as a pharmacological target because they are directly linked to cholesterol turnover [2,3] and also play an important role as signalling molecules in metabolic regulation [4,5]. The trial employed the NTCP inhibitor bulevirtide, which is known to increase systemic BA profoundly.

With NTCP inhibition, we investigated a novel approach for lipid-lowering, aiming at the intrahepatic consumption of LDL cholesterol and the consecutive upregulation of hepatic LDL receptors. Currently exploited pharmacological mechanisms for lowering LDL cholesterol include inhibition of cholesterol synthesis (statins, bempedoic acid) and inhibition of LDL receptor degradation (PCSK9 inhibitors, inclisiran) [23]. In addition, disruption of enterohepatic BA recirculation, which increases hepatocellular uptake of plasma LDL cholesterol for BA synthesis, is another pharmacological approach for the treatment of hyperlipidaemia; however, this is only so at the level of the intestinal resorption: BA-binding resins were key pharmacological agents in the early trials that proved the efficacy of decreasing LDL cholesterol in the prevention of cardiovascular events [20,24]. They act by blocking the reuptake of BA in the terminal ileum, but their use is limited by gastrointestinal side effects, including malabsorption of nutritional components and co-medication. With the targeted approach of blocking NTCP, we expected to induce plasma LDL-lowering effects at the level of the hepatocyte similar to those of the BA sequestrants while avoiding the intestinal adverse effects and drug interactions.

NTCP blockage was efficient, as demonstrated by a pronounced increase in circulating BA. The increase is in line with data on BA changes over several months from patients with hepatitis B/D treated with bulevirtide (unpublished data, Dr. Blank). In concordance with previous studies, conjugated BA rose more than unconjugated BA [21]; this is an observation also made in NTCP-deficient patients [25,26]. TCA can be considered a marker for the pharmacological effect of bulevirtide [21]. Good adherence to the IMP was confirmed by data on compliance from diaries and drug accounts and confirmed by pharmacokinetic measurements [22]. In lipid-lowering trials with high doses of cholestyramine, LDL cholesterol was lowered by 21% within 12 weeks [27]. However, in our 12-week trial of bulevirtide, we did not observe a statistically significant effect on LDL cholesterol. This may have several reasons:

Looking at individual data, it is apparent that the pharmacological response to bulevirtide differed. While seven participants showed a decrease in LDL cholesterol that was larger than the estimated effect size of 19.6 mg/dL, six participants showed less or no response. Although TCA trough concentrations rose in all participants as a result of bulevirtide treatment, in one individual, the level was clearly lower than in the others. Interestingly, this individual showed the highest LDL cholesterol level of all, both at baseline and after treatment. The impression that the effect on LDL cholesterol depends on the magnitude of the response to bulevirtide is supported by a negative correlation between LDL cholesterol and TCA trough concentrations. With the exception of the outlier described above, all individuals with minimal or no decrease in LDL cholesterol had a comparably low baseline LDL cholesterol. In conclusion, the LDL cholesterol-lowering effect of bulevirtide might depend on the baseline level of LDL cholesterol, given a sufficient pharmacological response to bulevirtide as measured by TCA.

The evasion of the cholesterol-lowering effect in some participants might be mitigated as follows: Even though NTCP is the main BA transporter, a fraction of circulating BA can enter the hepatocyte via other ways, such as OATP [18]. In the presence of elevated plasma BA, OATP expression might even be upregulated, as is the case in cholestatic liver disease, while NTCP is downregulated [28,29]. However, at least in vitro, OATP is also inhibited by bulevirtide [30], so this does not offer a sufficient explanation. BA transport via NTCP is specific for conjugated BA [5]. Moreover, OATP1B1 and OATP1B3 preferentially transport conjugated BA [31]. Unconjugated lipophilic BA, such as chenodeoxycholate, in contrast, is thought to also passively cross the cell membrane [32], being re-conjugated in the hepatocyte [5]. Thus, the effect of bulevirtide might be restricted to mainly blocking the active uptake of the conjugated BA, while the passive uptake of unconjugated BA is unaffected. This hypothesis is supported by the observation that plasma concentrations of conjugated BA are much more affected by bulevirtide than those of unconjugated BA. In conclusion, the NTCP-blocking effect of bulevirtide might be partially bypassed and therefore attenuated by the passive uptake of BA into the liver cell. Potential compensation mechanisms might also explain the temporary increase in LDL cholesterol after eight weeks.

In this exploratory trial, we describe the effect on thirteen volunteers, which is a limited number of participants. However, a mean change of 19.6 mg/dL with a standard deviation of 33.5 mg/dL in LDL cholesterol, as observed in this trial, could have been detected with a power of only ~48% in 13 patients (two-sided *t*-test, α = 0.05). Moreover, in contrast to most previous trials on lipid-lowering medications, the PrimaLiveR trial neither allowed to include hypercholesteraemic patients with clinically overt atherosclerotic disease (who would have been eligible for statin therapy) nor was standard lipid-lowering baseline therapy accepted. Therefore, a trial population was selected that was in a comparably good state of health and in an early stage of the atherosclerotic continuum, which may have made it more difficult to prove changes in predefined metabolic and inflammatory endpoints. The negative correlation between LDL cholesterol and TCA supports the description of an effect on LDL cholesterol and will be valuable as an estimation of effect size for further trials.

Regarding the secondary endpoints in lipid metabolism, there are two interesting observations indicating some effect on lipid metabolism: first, there was a statistically significant increase in HDL cholesterol after 12 weeks. This increase was, however, not consistent over the treatment period and thus could have been a statistical anomaly. It may still be cautiously interpreted as a however transient signal of a low level of FXR activation leading to this increase because, on the contrary, a decrease of HDL was observed in the pharmacological activation of FXR [9]. Furthermore, BA have been shown to reduce HDL endocytosis in human hepatocytes [32]. Second, there was a consistent but not statistically significant decrease in lipoprotein(a), which was reversible after the end of treatment. Lipoprotein(a) molar concentration is positively associated with an increased risk of coronary artery disease [33], and there is great interest in developing a lipoprotein(a) lowering medicine as no approved pharmacological treatment exists so far [34]. In patients with cholestasis, low plasma levels of lipoprotein(a) have been observed that were reversible upon removal of the obstruction, which has been explained by BA downregulating APOA transcription via the FXR-FGF15/19 pathway [35,36]. However, activation of this axis would imply an increase in LDL cholesterol and a decrease in HDL cholesterol, which was not the case in our trial [4]. Therefore, we cannot fully explain the statistically significant increase in HDL cholesterol and the not statistically significant decrease in lipoprotein(a) in the absence of a change in LDL cholesterol, which could also be a chance finding.

Concerning cardiac safety and tolerability, the trial can supplement the safety database for bulevirtide and BA. This is an important aspect of the trial as bulevirtide received accelerated conditional approval as an orphan disease medicine for hepatitis D. BA possess cardiotoxic properties. Interestingly, cardiotoxicity has been associated with the relative hydrophobicity of the BA pool [37]; however, bulevirtide increases the conjugated, hydrophilic BA more than it affects the unconjugated BA. CMR is considered the state-of-the-art imaging modality to detect cardiac toxicities as it possesses high accuracy and reproducibility in comparison to other diagnostic measures for the detection of adverse effects on cardiac structure and function and allows for more advanced tissue characterization than other imaging techniques [38]. In our study, T1 and T2 times and strains, which are all sensitive markers of myocardial composition and function allowing for the detection of even subtle impairments, were unchanged. We can conclude that in the study period, there were no signs of cardiotoxicity, neither from the IMP itself nor from elevated BA levels.

The small number of participants enrolled may have hindered us from detecting small biochemical changes. Despite all efforts, it was not possible to recruit a larger population of participants with substantial hypercholesterolemia without pre-existing (need for) established lipid-lowering therapy. This population is on the borderline between healthy volunteer participants and patients, a group of evolving interest in the process of turning away from a dichotomous understanding of cardiovascular health to the concept of an atherosclerotic risk continuum. Many screenings ended in forwarding applicants to established treatments for hyperlipidaemia, as the trial protocol was committed to avoiding the delay of required lipid-lowering therapy. Still, we would like to point out that this was an exploratory phase I trial; thus, by its nature, it intended to generate first information on the effect sizes—important information for calculating the sample size in later phase studies—and not to raise the claim of a final evaluation of efficacy.

At the time of the start of this trial, bulevirtide was not yet approved and was still under evaluation in phase II trials. The documented experience at this time was derived from a small number of hepatitis D patients with treatment durations of three to six months. In this situation, a treatment course of twelve weeks seemed appropriate for our trial and the exploratory approach. With regard to long-term endpoints, a twelve-week trial duration might be short, albeit most likely long enough for observing an LDL-cholesterol lowering effect, as is the case with other BA-modifying drugs [27], so we are convinced the trial duration is not a relevant limitation for the evaluation of the primary endpoint.

The strength of this exploratory study is the extensive diagnostic workup of the participants exposed to bulevirtide. We believe that our study adds valuable information to a pharmacological approach to cardiovascular prevention that remains to be fully understood. Authors of a recent review ask if “the benefits of NTCP inhibition seen in mice [can] be transferred to the human situation” [39], and we present the first answer. Our trial has generated the very first data on the effect sizes of important metabolic parameters from NTCP inhibition with bulevirtide, information that is indispensable for the planning of future trials in the field.

In conclusion, the estimated decrease in LDL cholesterol was 19.6 mg/dL after twelve weeks of treatment with bulevirtide. This was not statistically significant but described a potential effect size relevant for further research. On a mechanistic level, the cholesterol-lowering effect might be attenuated by passive BA uptake into the hepatocyte. We observed variation in the individual response. The described effect on LDL cholesterol—though not statistically significant—is supported by the observation of subtle changes in other lipid parameters and by the correlation of LDL cholesterol with the pharmacological inhibition of hepatocellular BA uptake as described by TCA plasma concentrations.

## 4. Methods

### 4.1. Study Design and Setting

PrimaLiveR was a single-centre, open-label, phase I pilot clinical study (EudraCT 2017-003137-28). The trial was approved by the responsible Ethics Committee of Heidelberg Medical Faculty (AFmo-670/2016) and by the competent authority (Federal Institute for Drugs and Medical Devices, BfArM, Bonn, Germany). There were two amendments to the study protocol. The first amendment was introduced to facilitate pre-screening procedures prior to a full screening for the trial. The second amendment was introduced to change the initial inclusion criteria for hypercholesteremia, which was LDL cholesterol > 200 mg/dL and total cholesterol > 200 mg/dL. The recruitment of this population with the requirement that no clinically relevant signs of atherosclerosis were allowed to be present was not reachable. The criteria were subsequently changed to (1) LDL cholesterol > 130 mg/dL and (2) total cholesterol > 200 mg/dL or total cholesterol > 180 mg/dL if LDL cholesterol was >160. The trial was conducted in the ISO9001-certified Early Clinical Trial Unit of the Department of Clinical Pharmacology and Pharmacoepidemiology at Heidelberg University Hospital, Germany, from May 2018 to December 2019 (last patient out). Written informed consent was obtained from every participant after extensive information before any trial procedures were initiated. We performed the trial according to the principles of Good Clinical Practice Guideline E6 and the Declaration of Helsinki.

### 4.2. Population

The main inclusion criteria were LDL cholesterol > 130 mg/dL (according to the amended study protocol; initially > 200 mg/dL) and a total cholesterol > 200 mg/dL (a total cholesterol of >180 mg/dL was accepted if LDL cholesterol was >160 mg/dL according to the amended study protocol). Men and non-pregnant/non-breastfeeding women aged 35 to 70 years were included if medical assessment did not reveal any acute clinically significant findings or any relevant abnormalities as determined by medical history, physical examination, clinical parameters (vital signs, electrocardiogram), and laboratory (hematology, biochemistry, urinalysis including drug screening test, human immunodeficiency virus (HIV) antibody screening test, and hepatitis B (HBV) or C virus screening test). The main cardiovascular exclusion criteria were concurrent medication interfering with lipid metabolism (e.g., statins), known familiar hypercholesterolemia, a SCORE ≥ 5% according to the 2016 guideline of the European Society of Cardiology [40], a history of cardiovascular disease, untreated diabetes mellitus, or uncontrolled hypertension. Treatment with antihypertensives (i.e., beta-blockers, ACE inhibitors, angiotensin receptor blockers, or calcium antagonists) and controlled diabetes mellitus were accepted if the medication regimen had been unchanged for the previous three months.

There was an optional pre-screening visit at which total cholesterol and LDL cholesterol levels could be measured if a documented lipid status was unavailable and hyperlipidaemia was only known to the patient from medical history.

### 4.3. Intervention

After the successful screening, participants were started to receive a once-daily 5 mg subcutaneous (s.c.) dose of bulevirtide for 12 weeks (Baccinex SA, Courroux, Switzerland), i.e., a dose higher than the currently approved s.c. dose of 2 mg/d. This had two reasons: the IC_50_ to block HBV infection is about 100 times lower than the IC_50_ required to block BA transport [13,15]. Because our study aimed to study the effect of increased BA, we chose the dose with the highest possible NTCP-blocking effect that had previously been proven to be safe (Bulevirtide had been approved in an accelerated process, where the dose with the most comprehensive safety data was approved, which was the 2 mg dose. Higher doses, such as the 5 mg/d and even 10 mg/d doses, are still under investigation and may be submitted for approval later).

The IMP was delivered in vials containing a lyophilized powder that was diluted in 1 mL of water for injection within two hours prior to administration. The IMP was stored at −20 °C. For home use, the vials were stored at +5 °C (±3 °C) (in the refrigerator); storage at room temperature was acceptable for up to three days. Participants were trained on the storage, reconstitution, and subcutaneous administration of the IMP. The participants noted every injection in a diary and brought all empty vials back to the study centre. This information, together with bile acid levels, was used to assess adherence. Participants were treated for 12 weeks and were regularly seen for assessment of adverse events and biosampling.

### 4.4. Endpoints

The primary endpoint was the difference in LDL cholesterol after 12 weeks of treatment with bulevirtide compared to baseline. Secondary endpoints were changes after 12 weeks of treatment compared to baseline in (1) lipid metabolism (total cholesterol, HDL cholesterol, non-HDL cholesterol, triglycerides, lipoprotein(a), apolipoprotein B, very low-density lipoprotein (VLDL)), (2) markers of endothelial function/inflammatory markers (hs-CRP; IL-1b, IL-6, IL-10, E-selectin, ICAM-1, TGF-β1, neopterin, and TNF-α), (3) myocardial function, myocardial tissue characterization (T1 and T2 time) and vascular function assessed by CMR, and (4) glucose metabolism (HOMA, HbA1c); the effect of bulevirtide on (5) BA (pre-dose trough, AUC, C_max_); (6) descriptive single-dose and steady-state bulevirtide pharmacokinetics (AUC, C_max_, T_max_, t_1/2_, and Cl); and (7) safety and tolerability of bulevirtide treatment.

### 4.5. Follow-Up Visits

Follow-up visits were performed after 1, 2, 4, 6, 8, 10, and 12 weeks of treatment. The end-of-trial visit took place one month after the end of treatment (EOT). Extended evaluations, including CMR, were carried out at baseline, after 12 weeks of treatment, and at the EOT visit. Pharmacokinetic assessments were performed after a single dose and at a steady state. After completion of the study, each participant received counselling from a cardiologist on the individual need for further primary cardiovascular preventive measures.

### 4.6. Assessments

#### 4.6.1. Lipid Profile

LDL cholesterol, total cholesterol, HDL cholesterol, triglycerides, lipoprotein(a), apolipoprotein B, and VLDL were measured in the accredited central laboratory of Heidelberg University Hospital, Germany, using ultracentrifugation.

#### 4.6.2. Biomarkers

hs-CRP was measured in the central laboratory of Heidelberg University Hospital (nephelometry). Pro- (IL-1ß, IL-6, TNF-α) and anti-inflammatory cytokines (IL-10, TGF-ß), as well as adhesion molecules (ICAM-1, E-selectin) and neopterin as an indicator of immune responses, were determined in the laboratory of the Institute of Immunology at Heidelberg University. IL-10 was below the lower limit of quantification in several patients at each visit (<0.234 pg/mL) and is therefore not presented in the results.

#### 4.6.3. Glucose Metabolism

HOMA was calculated from glucose and insulin (electrochemiluminescence immunoassay); HbA1c was measured using high-performance liquid chromatography (HPLC) at the central laboratory of Heidelberg University Hospital.

#### 4.6.4. Cardiac MRI

CMR scans included the assessment of the left ventricular dimensions, ejection fraction, global circumferential and longitudinal left ventricular strains, as well as T1 and T2 mapping. In addition, oxygenation-sensitive CMR was performed to assess changes in myocardial oxygenation. The baseline evaluation consisted of two CMRs (7 ± 1 d apart). A third single on-treatment CMR was performed at the completion of the 12-week treatment. A fourth post-treatment CMR took place at the end-of-trial visit one month later. CMR imaging and evaluation were performed in the Department of Cardiology, Pneumology and Angiology of Heidelberg University Hospital, Germany.

#### 4.6.5. Pharmacokinetics

Blood sampling, measurement, and analysis of bulevirtide with a validated ultra-performance liquid chromatography–tandem mass spectrometry assay were performed as described earlier [22].

#### 4.6.6. Bile Acids

BA measurements were performed by liquid chromatography quadrupole time-of-flight mass spectrometry (LC-qTOF) as described by Haag and colleagues [41].

#### 4.6.7. Safety Evaluation

All adverse events were assessed in terms of their seriousness, severity (terminology according to the then pertinent version of CTCAE (v4.3), and causality. Adverse events were coded using MedDRA versions 22.1 (English), 23.0, and 23.1.

A 12-lead ECG was performed at screening, after four, eight, and twelve weeks of treatment, and at end of the study. We performed a post hoc analysis of heart rate and the conduction intervals at screening and after 12 weeks of study treatment (Excel Microsoft Windows 10).

### 4.7. Statistics

Given the exploratory nature of the trial, there was no formal sample size calculation. The proposed sample size comprised 20 participants. The primary analysis as well as all other efficacy and pharmacokinetic (PK) analyses were performed in the per-protocol set, defined as all participants have received at least 80% of the study medication for at least ten weeks. For the primary analysis, missing values of the primary endpoint were replaced with multiple imputations using other humoral parameters from the same patient and visits, if available, or LDL cholesterol levels from past visits from the same patient, obtaining a chained recursive linear regression model estimating current from past measurements of patients. The Hodges–Lehmann estimator (=pseudo median) with asymptotic 95% confidence interval [42] was calculated as the location parameter of the 12-weeks-to-baseline difference and underlying values for the primary endpoint as well as for the parameters pertaining to lipid metabolism, markers of endothelial function, CMR, and glucose metabolism. No *p*-values were calculated because the main goal was to estimate the effects and their precisions. The changes from baseline are regarded as statistically significant if the 0 is not included in the respective 95%-confidence interval. For improved visual display of individual data, a post hoc grouping into “responders” (response in primary endpoint larger than the estimated effect size) and “non-responders” (response in primary endpoint smaller than the estimated effect size) was introduced. No tests were performed between these post hoc groups. For CMR, the baseline was defined as the mean values of the two baseline examinations. For BA, mean and standard deviation of trough, area under the curve (AUC), and maximum concentration (C_max_) were calculated. A posthoc analysis was performed for correlation between LDL cholesterol and TCA trough concentrations (Spearman’s rho). Pharmacokinetic parameters were calculated using Kinetica (Thermo Fisher Scientific, Waltham, MA, USA, version 5.0). For C_max_ and trough concentrations and for the AUC, mean and standard deviation were calculated.

The safety set was defined as all patients included in the trial and ever receiving study medication. As sensitivity analysis, the primary analysis was repeated on the safety population. In addition, the primary analysis for the parameters of lipid metabolism, markers of endothelial function, and glucose metabolism was repeated for the per-protocol set using the mean of the screening and baseline value instead of the baseline value.

Biometric analysis was defined in a statistical analysis plan that was authorized by the biometrician and the coordinating investigator. Statistical analyses were performed in SAS 9.4.

## Figures and Tables

**Table 1:** Characteristics of the trial participants at baseline (n = 14, safety population) ^1^.

Characteristic		
Age (years; mean [min; max])	57.3	[40; 64]
Gender		
Male	7	50%
Female	7	50%
Body mass index (BMI) (kg/m^2^; mean [min; max])	27.1	[21.3; 40.1]
Antihypertensives		
ACE inhibitors	2	14.3%
AT 1 antagonist	1	7.14%
Beta blocker	2	14.3%
HbA1c (mmol/mol)		
<39	8	57.1%
39–≤46 ^2^	6	42.9%
Smoking status		
Active smoker	4	28.6%
Past smoker	4	28.6%
Never smoker	6	42.9%

^1^ Due to rounding, totals may differ from 100%. ^2^ One participant (with known pre-diabetes and HbA1c of 46 mmol/mol) had an HbA1c of 48 mmol/mol at the first measurement.

**Table 2:** Baseline lipid profile and changes in lipid profile after 12 weeks of treatment with bulevirtide (5 mg/d; n = 13, per-protocol population). HL: Hodges–Lehmann, CI: Confidence interval.

	Pseudo Median (HL Estimate)	95% CI	Change (HL Estimate)	95% CI
Primary outcome:				
LDL cholesterol (mg/dL)	196	[176; 223]	−19.6	[−41.8; 2.85]
Secondary outcomes:				
Total cholesterol (mg/dL)	270	[251; 295]	−7	[−30.5; 15.0]
HDL cholesterol (mg/dL)	62	[54.0; 72.0]	5.5	[1.00; 10.5]
VLDL cholesterol (mg/dL)	10.7	[5.60; 15.8]	2.4	[−6.30; 19.1]
Non-HDL (mg/dL)	206	[181; 237]	−8	[−30.0; 17.0]
Triglycerides (mg/dL)	118	[93.5; 141]	−17	[−35.0; 5.0]
Apolipoprotein B (g/L)	1.25	[1.14; 1.43]	−0.045	[−0.19; 0.11]
Lipoprotein(a) (mg/dL)	15.0	[10.1; 68.1]	−1.87	[−7.65; 0.00]

**Table 3:** Baseline parameters and changes in inflammatory markers, myocardial function, tissue mapping, and glucose metabolism after 12 weeks of treatment with bulevirtide (5 mg/d; n = 13, per-protocol population). HL: Hodges–Lehmann, CI: Confidence interval.

	Pseudo Median (HL Estimate)	95% CI	Change (HL Estimate)	95% CI
hs-CRP (mg/L)	1.06	[0.66; 1.78]	−0.055	[−0.22; 0.80]
IL-1b (pg/mL)	0.12	[0.10; 0.15]	0.003	[−0.02; 0.05]
IL-6 (pg/mL)	1.01	[0.68; 1.55]	−0.054	[−0.33; 0.13]
TNF-α (pg/mL)	7.64	[6.09; 9.19]	0.167	[−0.24; 0.61]
E-selectin (pg/mL)	32,654	[28,403; 37,045]	−184	[−2369; 2476]
ICAM-1 (pg/mL)	295,745	[249,257; 359,690]	−4158	[−17,457; 13,677]
TGF-β1 (pg/mL)	11,810	[9420; 14,071]	−493	[−3830; 4265]
Neopterin (µmol/L)	8.08	[7.17; 9.00]	0.095	[−0.61; 0.72]
LVEF (%)	62.8	[60.8; 64.5]	−0.25	[−1.75; 1.00]
global circumferential strain (%)	−17.7	[−18.8; −16.5]	−0.2	[−1.48; 1.57]
global longitudinal strain (%)	−13.7	[−14.9; −12.4]	0.05	[−1.74; 1.15]
global T1 time (ms)	1236	[1221; 1254]	13.8	[−0.26; 35.8]
global T2 time (ms)	46.3	[45.5; 47.9]	1.37 *	[−0.43; 2.97] *
HbA1c (mmol/mol)	38	[34; 41]	−0.5	[−3.50; 1.50]
HOMA	2.15	[1.19; 2.96]	−0.01	[−0.35; 0.25]

* value of one patient was missing.

## Data Availability

The data presented in this study are available on request from the corresponding author.

## Supplementary Materials

The following supporting information can be downloaded at: https://www.mdpi.com/article/10.3390/ijms232415924/s1.
