# Supplementary material for: Metabolic Effect of Blocking Sodium-Taurocholate Co-Transporting Polypeptide in Hypercholesterolemic Humans with a Twelve-Week Course of Bulevirtide—An Exploratory Phase I Clinical Trial
Source: Int J Mol Sci. 2022 Dec 14;23(24):15924. doi: 10.3390/ijms232415924 (PMC9787649; doi:10.3390/ijms232415924)
Supplement: Supplementary file 1 [file ijms-23-15924-s001.zip › ijms-2014857-supplementary.pdf]

Supplementary Material

1 Lipid profiles (per-protocol population)

LDL – uc, mg/dl

	Visit	Parameter	n	HL-estimate	95%-CI	Minimum	25%-Quantile	Median	75%-Quantile	Maximum
Total (N=13)	Baseline	LDL – uc, mg/dl	13	196	[176; 223]	157	167	192	224	279
	Visit W2D1	LDL – uc, mg/dl	13	185	[168; 218]	144	164	193	207	295
	Visit W5D1	LDL – uc, mg/dl	13	183	[161; 207]	139	161	167	211	252
	Visit W9D1	LDL – uc, mg/dl	13	198	[173; 221]	123	172	201	220	266
	Visit W13D1	LDL – uc, mg/dl	13	178	[156; 202]	126	150	178	193	277
	End of study visit	LDL – uc, mg/dl	13	192	[175; 216]	138	176	186	216	288

LDL – uc, mg/dl - change

	Visit	Parameter	n	HL-estimate	95%-CI	Minimum	25%-Quantile	Median	75%-Quantile	Maximum
Total (N=13)	Visit W2D1	LDL – uc, mg/dl	13	-9.1	[-27.0; 9.9]	-47.2	-23.4	-21.3	6.8	45.3
	Visit W5D1	LDL – uc, mg/dl	13	-12.6	[-31.2; 5.0]	-84.6	-30.7	-10.7	5.5	36.9
	Visit W9D1	LDL – uc, mg/dl	13	-3.4	[-28.4; 22.2]	-71.9	-28.9	3.3	21.6	79.1

LDL – uc, mg/dl - change

	Visit	Parameter	n	HL-estimate	95%-CI	Minimum	25%-Quantile	Median	75%-Quantile	Maximum
	Visit W13D1	LDL – uc, mg/dl	13	-19.6	[-41.8; 2.9]	-73.8	-41.8	-21.7	-2.1	34.6
	End of study visit	LDL – uc, mg/dl	13	-2.2	[-23.6; 16.3]	-56.7	-15.8	7.2	18.5	34.6

Total Cholesterol- uc, mg/dl

	Visit	Parameter	n	HL-estimate	95%-CI	Minimum	25%-Quantile	Median	75%-Quantile	Maximum
Total (N=13)	Baseline	Total Cholesterol-uc, mg/dl	13	270	[251; 295]	243	251	261	292	350
	Visit W2D1	Total Cholesterol-uc, mg/dl	13	273	[253; 297]	228	243	278	292	359
	Visit W5D1	Total Cholesterol-uc, mg/dl	13	269	[242; 294]	199	241	254	300	336
	Visit W9D1	Total Cholesterol-uc, mg/dl	13	276	[251; 299]	170	257	279	299	335
	Visit W13D1	Total Cholesterol-uc, mg/dl	13	265	[237; 295]	189	246	262	283	349
	End of study visit	Total Cholesterol-uc, mg/dl	13	276	[251; 305]	199	257	265	303	364

Total Cholesterol- uc, mg/dl - change

	Visit	Parameter	n	HL-estimate	95%-CI	Minimum	25%-Quantile	Median	75%-Quantile	Maximum
Total (N=13)	Visit W2D1	Total Cholesterol-uc, mg/dl	13	1.5	[-15.0; 20.0]	-49	-17	-1	20	43
	Visit W5D1	Total Cholesterol-uc, mg/dl	13	-7	[-24.0; 17.0]	-81	-22	-10	8	58
	Visit W9D1	Total Cholesterol-uc, mg/dl	13	2	[-21.5; 18.5]	-81	-10	9	18	59
	Visit W13D1	Total Cholesterol-uc, mg/dl	13	-7	[-30.5; 15.0]	-73	-26	-1	23	42
	End of study visit	Total Cholesterol-uc, mg/dl	13	7	[-21.0; 23.5]	-60	1	8	18	42

HDL – uc, mg/dl

	Visit	Parameter	n	HL-estimate	95%-CI	Minimum	25%-Quantile	Median	75%-Quantile	Maximum
Total (N=13)	Baseline	HDL – uc, mg/dl	13	62	[54.0; 72.0]	42	54	56	70	96
	Visit W2D1	HDL – uc, mg/dl	13	64	[56.0; 76.5]	49	55	61	69	100
	Visit W5D1	HDL – uc, mg/dl	13	64	[57.5; 77.0]	50	58	64	72	128
	Visit W9D1	HDL – uc, mg/dl	13	61	[53.5; 73.0]	45	54	59	67	91
	Visit W13D1	HDL – uc, mg/dl	13	66.5	[59.5; 77.5]	49	58	64	71	100

HDL – uc, mg/dl

	Visit	Parameter	n	HL-estimate	95%-CI	Minimum	25%-Quantile	Median	75%-Quantile	Maximum
	End of study visit	HDL – uc, mg/dl	13	66.5	[53.0; 79.5]	43	52	58	77	107

HDL – uc, mg/dl - change

	Visit	Parameter	n	HL-estimate	95%-CI	Minimum	25%-Quantile	Median	75%-Quantile	Maximum
Total (N=13)	Visit W2D1	HDL – uc, mg/dl	13	3	[-0.5; 7.0]	-7	0	2	6	13
	Visit W5D1	HDL – uc, mg/dl	13	5.5	[-2.0; 12.0]	-15	3	4	8	32
	Visit W9D1	HDL – uc, mg/dl	13	-0.5	[-6.5; 7.5]	-29	-6	-2	6	23
	Visit W13D1	HDL – uc, mg/dl	13	5.5	[1.0; 10.5]	-9	2	5	9	23
	End of study visit	HDL – uc, mg/dl	13	4	[-1.0; 9.5]	-8	1	4	7	27

VLDL - uc, mg/dl

	Visit	Parameter	n	HL-estimate	95%-CI	Minimum	25%-Quantile	Median	75%-Quantile	Maximum
Total (N=13)	Baseline	VDL - uc, mg/dl	13	10.7	[5.60; 15.8]	0.9	3.5	11	16	25.5
	Visit W2D1	VDL - uc, mg/dl	13	13.1	[7.40; 26.0]	2.6	7.4	9.9	17.1	70.5
	Visit W5D1	VDL - uc, mg/dl	13	13.3	[8.50; 17.5]	3.9	8.4	11.7	20	24.9
	Visit W9D1	VDL - uc, mg/dl	13	11.4	[8.20; 16.3]	2.4	8.2	10.9	16.3	26.8

VLDL - uc, mg/dl

	Visit	Parameter	n	HL-estimate	95%-CI	Minimum	25%-Quantile	Median	75%-Quantile	Maximum
	Visit W13D1	VDL - uc, mg/dl	13	11.9	[6.15; 26.1]	2.2	4.5	10	15.2	73.4
	End of study visit	VDL - uc, mg/dl	13	11.9	[5.95; 20.9]	1.1	5.6	8.9	22.6	35.4

VLDL - uc, mg/dl - change

	Visit	Parameter	n	HL-estimate	95%-CI	Minimum	25%-Quantile	Median	75%-Quantile	Maximum
Total (N=13)	Visit W2D1	VDL - uc, mg/dl	13	3.9	[-3.80; 17.90]	-16.3	-3.6	3	13.4	54.5
	Visit W5D1	VDL - uc, mg/dl	13	2.15	[-1.65; 6.70]	-5.5	-4.4	3.7	6.1	17
	Visit W9D1	VDL - uc, mg/dl	13	0.4	[-6.10; 8.70]	-23	-4.1	0.4	8.9	23.7
	Visit W13D1	VDL - uc, mg/dl	13	2.4	[-6.30; 19.10]	-12.2	-7.3	-2.3	12.7	52.6
	End of study visit	VDL - uc, mg/dl	13	0.45	[-4.65; 9.90]	-10.4	-4.7	0.7	5.4	29.8

Non-HDL - uc, mg/dl

	Visit	Parameter	n	HL-estimate	95%-CI	Minimum	25%-Quantile	Median	75%-Quantile	Maximum
Total (N=13)	Baseline	Non-HDL - uc, mg/dl	13	206	[181; 237]	96	187	198	238	296
	Visit W2D1	Non-HDL - uc, mg/dl	13	206	[185; 231]	153	179	214	229	304
	Visit W5D1	Non-HDL - uc, mg/dl	13	195	[176; 223]	149	174	187	219	277

Non-HDL - uc, mg/dl

	Visit	Parameter	n	HL-estimate	95%-CI	Minimum	25%-Quantile	Median	75%-Quantile	Maximum
	Visit W9D1	Non-HDL - uc, mg/dl	13	213	[184; 234]	125	181	217	234	276
	Visit W13D1	Non-HDL - uc, mg/dl	13	196	[170; 225]	131	164	192	212	286
	End of study visit	Non-HDL - uc, mg/dl	13	206	[185; 221]	142	187	209	221	245

Non-HDL - uc, mg/dl - change

	Visit	Parameter	n	HL-estimate	95%-CI	Minimum	25%-Quantile	Median	75%-Quantile	Maximum
Total (N=13)	Visit W2D1	Non-HDL - uc, mg/dl	13	0.5	[-17.0; 21.0]	-59	-17	0	11	108
	Visit W5D1	Non-HDL - uc, mg/dl	13	-10.5	[-27.0; 17.5]	-89	-24	-13	3	95
	Visit W9D1	Non-HDL - uc, mg/dl	13	3.5	[-25.5; 35.0]	-73	-29	7	27	92
	Visit W13D1	Non-HDL - uc, mg/dl	13	-8	[-30.0; 17.0]	-86	-29	-9	21	50
	End of study visit	Non-HDL - uc, mg/dl	13	-2.5	[-30.5; 22.5]	-74.9	-25	1	14	100

Triglycerides- uc, mg/dl

	Visit	Parameter	n	HL-estimate	95%-CI	Minimum	25%-Quantile	Median	75%-Quantile	Maximum
Total (N=13)	Baseline	Triglycerides-uc, mg/dl	13	118	[93.5; 141]	39	97	120	139	192
	Visit W2D1	Triglycerides-uc, mg/dl	13	118	[95.0; 162]	45	93	111	140	231

Triglycerides- uc, mg/dl

	Visit	Parameter	n	HL-estimate	95%-CI	Minimum	25%-Quantile	Median	75%-Quantile	Maximum
	Visit W5D1	Triglycerides-uc, mg/dl	13	112	[92.0; 134]	60	92	102	137	176
	Visit W9D1	Triglycerides-uc, mg/dl	13	123	[102.5; 142]	50	105	124	135	178
	Visit W13D1	Triglycerides-uc, mg/dl	13	100	[78.5; 127]	56	69	93	128	168
	End of study visit	Triglycerides-uc, mg/dl	13	102	[81.5; 146]	50	82	95	122	230

apolipoprotein B - uc, g/l

	Visit	Parameter	n	HL-estimate	95%-CI	Minimum	25%-Quantile	Median	75%-Quantile	Maximum
Total (N=13)	Baseline	apolipo-protein B - uc, g/l	13	1.25	[1.14; 1.43]	1.01	1.14	1.23	1.35	1.98
	Visit W2D1	apolipoprotein B - uc, g/l	13	1.27	[1.14; 1.40]	0.96	1.16	1.29	1.33	1.99
	Visit W5D1	apolipoprotein B - uc, g/l	13	1.20	[1.09; 1.39]	0.92	1.09	1.18	1.33	1.72
	Visit W9D1	apolipoprotein B - uc, g/l	13	1.27	[1.13; 1.44]	0.97	1.11	1.28	1.42	1.77

apolipoprotein B - uc, g/l

	Visit	Parameter	n	HL-estimate	95%-CI	Minimum	25%-Quantile	Median	75%-Quantile	Maximum
	Visit W13D1	apolipoprotein B - uc, g/l	13	1.21	[1.09; 1.39]	0.9	1.02	1.2	1.37	1.87
	End of study visit	apolipoprotein B - uc, g/l	13	1.23	[1.10; 1.38]	0.88	1.08	1.21	1.33	1.99

apolipoprotein B - uc, g/l – change

	Visit	Parameter	n	HL-estimate	95%-CI	Minimum	25%-Quantile	Median	75%-Quantile	Maximum
Total (N=13)	Visit W2D1	apolipoprotein B - uc, g/l	13	-0.01	[-0.06; 0.06]	-0.18	-0.06	-0.02	0.01	0.17
	Visit W5D1	apolipoprotein B - uc, g/l	13	-0.05	[-0.14; 0.06]	-0.3	-0.12	-0.05	0.06	0.24
	Visit W9D1	apolipoprotein B - uc, g/l	13	-0.02	[-0.14; 0.11]	-0.32	-0.17	-0.01	0.05	0.51
	Visit W13D1	apolipoprotein B - uc, g/l	13	-0.045	[-0.19; 0.11]	-0.4	-0.21	-0.1	0.16	0.31

apolipoprotein B - uc, g/l – change

	Visit	Parameter	n	HL-estimate	95%-CI	Minimum	25%-Quantile	Median	75%-Quantile	Maximum
	End of study visit	apolipoprotein B - uc, g/l	13	-0.005	[-0.17; 0.08]	-0.42	-0.06	0.01	0.08	0.31

lipoprotein (a) - uc, mg/dl

	Visit	Parameter	n	HL-estimate	95%-CI	Minimum	25%-Quantile	Median	75%-Quantile	Maximum
Total (N=13)	Baseline	lipoprotein (a) - uc, mg/dl	13	15	[10.1; 68.1]	10	10	12.9	19.9	163
	Visit W2D1	lipoprotein (a) - uc, mg/dl	13	14.4	[10.0; 48.2]	10	10	11.3	18.7	117
	Visit W5D1	lipoprotein (a) - uc, mg/dl	13	14.3	[10.0; 54.5]	10	10	10	18.6	124
	Visit W9D1	lipoprotein (a) - uc, mg/dl	13	17.4	[10.0; 57.3]	10	10	10	24.7	136
	Visit W13D1	lipoprotein (a) - uc, mg/dl	13	16.3	[10.0; 60.4]	10	10	10.1	22.6	111

lipoprotein (a) - uc, mg/dl

	Visit	Parameter	n	HL-estimate	95%-CI	Minimum	25%-Quantile	Median	75%-Quantile	Maximum
	End of study visit	lipoprotein (a) - uc, mg/dl	13	16.3	[10.3; 68.4]	10	10	12.9	22.6	127

lipoprotein (a) - uc, mg/dl - change

	Visit	Parameter	n	HL-estimate	95%-CI	Minimum	25%-Quantile	Median	75%-Quantile	Maximum
Total (N=13)	Visit W2D1	lipoprotein (a) - uc, mg/dl	13	-1.36	[-4.27; -0.05]	-78.5	-2.72	-0.82	0	0
	Visit W5D1	lipoprotein (a) - uc, mg/dl	13	-2	[-13.04; 0.00]	-38.8	-4	-0.11	0	3.51
	Visit W9D1	lipoprotein (a) - uc, mg/dl	13	-2	[-10.26; 0.00]	-26.9	-4	-0.11	0	7.68
	Visit W13D1	lipoprotein (a) - uc, mg/dl	13	-1.87	[-7.65; 0.00]	-51.4	-3.74	-0.11	0	2.68
	End of study visit	lipoprotein (a) - uc, mg/dl	13	0.19	[-1.35; 2.68]	-35.9	0	0	2.68	8.46

2 Inflammatory biomarkers (per-protocol population)

hs-CRP, mg/l

	Visit	Parameter	n	HL-estimate	95%-CI	Minimum	25%-Quantile	Median	75%-Quantile	Maximum
Total (N=13)	Baseline	hs-CRP, mg/l	13	1.06	[0.66; 1.78]	0.2	0.67	0.98	1.74	6.63
	Visit W2D1	hs-CRP, mg/l	13	1.09	[0.77; 1.58]	0.32	0.71	1	1.4	2.72
	Visit W5D1	hs-CRP, mg/l	13	1.435	[0.77; 5.18]	0.19	0.82	1.14	2.68	11.9
	Visit W9D1	hs-CRP, mg/l	13	1.05	[0.59; 1.55]	0.23	0.46	0.95	1.74	2.67
	Visit W13D1	hs-CRP, mg/l	13	1.11	[0.47; 1.93]	0.23	0.47	0.62	1.76	3.39
	End of study visit	hs-CRP, mg/l	12	0.943	[0.47; 1.64]	0.18	0.435	0.775	1.51	8.88

hs-CRP, mg/l - change

	Visit	Parameter	n	HL-estimate	95%-CI	Minimum	25%-Quantile	Median	75%-Quantile	Maximum
Total (N=13)	Visit W2D1	hs-CRP, mg/l	13	0.065	[-0.23; 0.42]	-5.63	-0.15	0.06	0.42	0.98
	Visit W5D1	hs-CRP, mg/l	13	0.185	[-0.32; 4.08]	-5.29	-0.23	-0.01	0.87	11.4
	Visit W9D1	hs-CRP, mg/l	13	-0.01	[-0.30; 0.45]	-6.09	-0.21	0.03	0.15	1.36
	Visit W13D1	hs-CRP, mg/l	13	-0.055	[-0.22; 0.80]	-6.05	-0.18	-0.05	0.03	2.27
	End of study visit	hs-CRP, mg/l	12	-0.135	[-0.30; 0.23]	-0.8	-0.255	-0.145	0.065	7.86

IL-10, pg/ml

	Visit	Parameter	n	HL-estimate	95%-CI	Minimum	25%-Quantile	Median	75%-Quantile	Maximum
Total (N=13)	Baseline	IL-10, pg/ml	10	0.234	[0.23; 0.41]	0.234	0.234	0.234	0.234	0.576
	Visit W2D1	IL-10, pg/ml	6	0.234	[0.23; 0.29]	0.234	0.234	0.234	0.234	0.353
	Visit W5D1	IL-10, pg/ml	13	0.234	[0.23; 0.33]	0.204	0.234	0.234	0.234	0.622
	Visit W9D1	IL-10, pg/ml	12	0.334	[0.23; 0.52]	0.0817	0.234	0.234	0.482	1.19
	Visit W13D1	IL-10, pg/ml	8	0.234	[0.22; 0.58]	0.204	0.234	0.234	0.234	0.932
	End of study visit	IL-10, pg/ml	11	0.234	[0.23; 0.29]	0.234	0.234	0.234	0.234	0.350

IL-10, pg/ml - change

	Visit	Parameter	n	HL-estimate	95%-CI	Minimum	25%-Quantile	Median	75%-Quantile	Maximum
Total (N=13)	Visit W2D1	IL-10, pg/ml	6	0	[-0.11; 0.00]	-0 .224	0	0	0	0
	Visit W5D1	IL-10, pg/ml	10	0	[-0.07; 0.02]	-0 .149	0	0	0	0.036
	Visit W9D1	IL-10, pg/ml	9	0	[-0.07; 0.48]	-0 .133	0	0	0	0.952
	Visit W13D1	IL-10, pg/ml	6	0	[0.00; 0.18]	0	0	0	0	0.356
	End of study visit	IL-10, pg/ml	9	0	[0.00; 0.06]	0	0	0	0	0.116

IL1-b, pg/ml

	Visit	Parameter	n	HL-estimate	95%-CI	Minimum	25%-Quantile	Median	75%-Quantile	Maximum

IL1-b, pg/ml

	Visit	Parameter	n	HL-estimate	95%-CI	Minimum	25%-Quantile	Median	75%-Quantile	Maximum
Total (N=13)	Baseline	IL1-b, pg/ml	13	0.1227	[0.10; 0.15]	0.080	0.101	0.120	0.144	0.231
	Visit W2D1	IL1-b, pg/ml	13	0.125	[0.10; 0.17]	0.080	0.101	0.101	0.149	0.280
	Visit W5D1	IL1-b, pg/ml	13	0.120	[0.10; 0.14]	0.038	0.101	0.120	0.133	0.200
	Visit W9D1	IL1-b, pg/ml	13	0.130	[0.11; 0.24]	0.060	0.110	0.1227	0.140	0.718
	Visit W13D1	IL1-b, pg/ml	13	0.126	[0.11; 0.15]	0.100	0.120	0.1227	0.144	0.329
	End of study visit	IL1-b, pg/ml	13	0.123	[0.10; 0.14]	0.0381	0.100	0.1227	0.144	0.179

IL1-b, pg/ml - change

	Visit	Parameter	n	HL-estimate	95%-CI	Minimum	25%-Quantile	Median	75%-Quantile	Maximum

IL1-b, pg/ml - change

	Visit	Parameter	n	HL-estimate	95%-CI	Minimum	25%-Quantile	Median	75%-Quantile	Maximum
Total (N=13)	Visit W2D1	IL1-b, pg/ml	13	0	[-0.03; 0.05]	-0.129	-0.020	0	0.010	0.161
	Visit W5D1	IL1-b, pg/ml	13	-0.005	[-0.04; 0.03]	-0.129	-0.040	0	0.030	0.060
	Visit W9D1	IL1-b, pg/ml	13	0.005	[-0.04; 0.14]	-0.108	-0.043	0.010	0.040	0.617
	Visit W13D1	IL1-b, pg/ml	13	0.003	[-0.02; 0.05]	-0.087	-0.020	0	0.040	0.185
	End of study visit	IL1-b, pg/ml	13	-0.000	[-0.05; 0.03]	-0.151	-0.043	0.020	0.030	0.086

IL-6, pg/ml

	Visit	Parameter	n	HL-estimate	95%-CI	Minimum	25%-Quantile	Median	75%-Quantile	Maximum
Total (N=13)	Baseline	IL-6, pg/ml	13	1.01	[0.68; 1.55]	0.222	0.727	0.799	1.52	2.59
	Visit W2D1	IL-6, pg/ml	13	1.01	[0.72; 1.29]	0.143	0.804	1.0407361047	1.20	1.85
	Visit W5D1	IL-6, pg/ml	13	1.19	[0.72; 2.13]	0.102	0.702	0.969	1.57	4.93
	Visit W9D1	IL-6, pg/ml	13	0.993	[0.76; 1.18]	0.301	0.804	1.031700598	1.21	1.52
	Visit W13D1	IL-6, pg/ml	13	0.832	[0.59; 1.25]	0.262	0.604	0.746	1.06	1.94
	End of study visit	IL-6, pg/ml	13	0.949	[0.65; 1.27]	0.242	0.653	0.880	1.15	1.80

IL-6, pg/ml - change

	Visit	Parameter	n	HL-estimate	95%-CI	Minimum	25%-Quantile	Median	75%-Quantile	Maximum
Total (N=13)	Visit W2D1	IL-6, pg/ml	13	0.047	[-0.35; 0.29]	-2.19	-0.115	0.038	0.284	0.667
	Visit W5D1	IL-6, pg/ml	13	0.022	[-0.31; 1.07]	-1.02	-0.302	0	0.345	4.13
	Visit W9D1	IL-6, pg/ml	13	0.021	[-0.50; 0.27]	-2.04	-0.484	0.079	0.267	0.568
	Visit W13D1	IL-6, pg/ml	13	-0.054	[-0.33; 0.13]	-2.04	-0.282	-0.000	0.049	0.424
	End of study visit	IL-6, pg/ml	13	-0.008	[-0.31; 0.19]	-1.99	-0.188	0.020	0.242	0.418

TNF-a, pg/ml

	Visit	Parameter	n	HL-estimate	95%-CI	Minimum	25%-Quantile	Median	75%-Quantile	Maximum
Total (N=13)	Baseline	TNF-a, pg/ml	13	7.64	[6.09; 9.19]	3.63	5.73	8.17	9.05	13 .9
	Visit W2D1	TNF-a, pg/ml	13	7.89	[6.26; 9.65]	3.90	5.41	8.09	9.7	13
	Visit W5D1	TNF-a, pg/ml	13	7.91	[6.02; 9.66]	2.66	5.01	8.21	10 .1	12 .4
	Visit W9D1	TNF-a, pg/ml	13	8.09	[6.46; 9.76]	4.43	5.92	8.58	10 .4	14 .2
	Visit W13D1	TNF-a, pg/ml	13	7.78	[6.09; 9.65]	3.97	5.92	7.53	9.9	13 .4
	End of study visit	TNF-a, pg/ml	13	7.24	[5.77; 9.35]	3.01	5.545	7.05	9.35	14 .7

TNF-a, pg/ml - change

	Visit	Parameter	n	HL-estimate	95%-CI	Minimum	25%-Quantile	Median	75%-Quantile	Maximum
Total (N=13)	Visit W2D1	TNF-a, pg/ml	13	0.179	[-0.22; 0.53]	-0 .902	-0 .241	0.269	0.596	0.953
	Visit W5D1	TNF-a, pg/ml	13	-0 .201	[-0.76; 1.06]	-1 .479	-0 .683	-0 .281	0.307	3.66
	Visit W9D1	TNF-a, pg/ml	13	0.386	[0.06; 0.80]	-0 .566	0.190	0.266	0.677	1.53
	Visit W13D1	TNF-a, pg/ml	13	0.167	[-0.24; 0.61]	-0 .687	-0 .483	0.200	0.528	1.54
	End of study visit	TNF-a, pg/ml	13	-0 .119	[-0.52; 0.31]	-1 .646	-0 .310	-0 .120	0.134	1.02

E-Selectin, pg/ml

	Visit	Parameter	n	HL-estimate	95%-CI	Minimum	25%-Quantile	Median	75%-Quantile	Maximum
Total (N=13)	Baseline	E-Selectin, pg/ml	13	32654	[28403; 37045]	21301	28403	33179	37693	44370
	Visit W2D1	E-Selectin, pg/ml	13	34049	[28828; 38839]	23984	28377	31022	40356	45264
	Visit W5D1	E-Selectin, pg/ml	13	31457	[26675; 36449]	18417	25820	31359	36808	47153
	Visit W9D1	E-Selectin, pg/ml	13	33829	[29174; 37892]	18161	30480	33573	39665	43958
	Visit W13D1	E-Selectin, pg/ml	13	32428	[27404; 36822]	18948	26573	32248	37676	47068
	End of study visit	E-Selectin, pg/ml	13	31473	[26702; 36835]	20707	26035	31699	34568	44878

E-Selectin, pg/ml - change

	Visit	Parameter	n	HL-estimate	95%-CI	Minimum	25%-Quantile	Median	75%-Quantile	Maximum
Total (N=13)	Visit W2D1	E-Selectin, pg/ml	13	1216	[-1203; 4056]	-5068	-1635	893	3562	9804
	Visit W5D1	E-Selectin, pg/ml	13	-1628	[-4405; 2798]	-8378	-5003	-1248	8.26	13973
	Visit W9D1	E-Selectin, pg/ml	13	568	[-2279; 4000]	-7800	-3297	2751	4000	8423
	Visit W13D1	E-Selectin, pg/ml	13	-184	[-2369; 2476]	-8480	-2282	-595	2698	5883
	End of study visit	E-Selectin, pg/ml	13	-731	[-3254; 1254]	-10100	-3155	-594	508	4359

ICAM-1, pg/ml

	Visit	Parameter	n	HL-estimate	95%-CI	Minimum	25%-Quantile	Median	75%-Quantile	Maximum

ICAM-1, pg/ml

	Visit	Parameter	n	HL-estimate	95%-CI	Minimum	25%-Quantile	Median	75%-Quantile	Maximum
Total (N=13)	Baseline	ICAM-1, pg/ml	13	295745	[249257; 359690]	190969	247968	290030	344219	633252
	Visit W2D1	ICAM-1, pg/ml	13	289332	[250086; 360124]	197403	250895	279724	334920	631535
	Visit W5D1	ICAM-1, pg/ml	13	287657	[248329; 351460]	205766	249023	278724	321011	586698
	Visit W9D1	ICAM-1, pg/ml	13	296833	[253506; 380812]	177639	243245	284114	342623	583985
	Visit W13D1	ICAM-1, pg/ml	13	303129	[242982; 386021]	171457	239024	293583	325718	613146
	End of study visit	ICAM-1, pg/ml	13	294410	[242265; 388603]	179097	232113	284627	341190	608310

ICAM-1, pg/ml - change

	Visit	Parameter	n	HL-estimate	95%-CI	Minimum	25%-Quantile	Median	75%-Quantile	Maximum

ICAM-1, pg/ml - change

	Visit	Parameter	n	HL-estimate	95%-CI	Minimum	25%-Quantile	Median	75%-Quantile	Maximum
Total (N=13)	Visit W2D1	ICAM-1, pg/ml	13	-4084	[-13243; 3732]	-28199	-10307	-4478	2990	16602
	Visit W5D1	ICAM-1, pg/ml	13	-13293	[-26415; 2673]	-46554	-24919	-15281	1055	30265
	Visit W9D1	ICAM-1, pg/ml	13	-373	[-13638; 13003]	-49267	-13331	-1596	12783	44441
	Visit W13D1	ICAM-1, pg/ml	13	-4158	[-17457; 13677]	-33972	-19512	-5201	628	102801
	End of study visit	ICAM-1, pg/ml	13	-7871	[-18500; 6373]	-38786	-18500	-5896	1538	100720

TGF-b1, pg/ml

	Visit	Parameter	n	HL-estimate	95%-CI	Minimum	25%-Quantile	Median	75%-Quantile	Maximum
Total (N=13)	Baseline	TGF-b1, pg/ml	13	11810	[9420; 14071]	7547	9269	10950	14692	17797
	Visit W2D1	TGF-b1, pg/ml	12	12796	[9951; 14948]	7739	9863	11912	15839	18919
	Visit W5D1	TGF-b1, pg/ml	13	10145	[8792; 11735]	6976	8678	9594	11698	15020
	Visit W9D1	TGF-b1, pg/ml	13	11057	[9317; 13025]	7860	8964	9940	12489	66374
	Visit W13D1	TGF-b1, pg/ml	13	10924	[9059; 14813]	6759	9644	10217	13250	32520
	End of study visit	TGF-b1, pg/ml	13	11778	[9563; 14334]	5610	8896	10882	14666	17946

TGF-b1, pg/ml - change

	Visit	Parameter	n	HL-estimate	95%-CI	Minimum	25%-Quantile	Median	75%-Quantile	Maximum
Total (N=13)	Visit W2D1	TGF-b1, pg/ml	12	1007	[-1021; 3639]	-5325	-1021	948	3486	9650
	Visit W5D1	TGF-b1, pg/ml	13	-1122	[-3815; 946]	-9348	-3194	-1697	1426	3013
	Visit W9D1	TGF-b1, pg/ml	13	-1034	[-3511; 1925]	-7900	-2310	-1034	1152	58826
	Visit W13D1	TGF-b1, pg/ml	13	-493	[-3830; 4265]	-8125	-4516	-526	2926	21816
	End of study visit	TGF-b1, pg/ml	13	311	[-2036; 2361]	-7074	-2036	458	947	7118

Neopterin, µmol/l

	Visit	Parameter	n	HL-estimate	95%-CI	Minimum	25%-Quantile	Median	75%-Quantile	Maximum
Total (N=13)	Baseline	Neopterin, µmol/l	13	8.08	[7.17; 9.00]	6.14	7.16	8.06	9	10.1
	Visit W2D1	Neopterin, µmol/l	13	7.92	[7.06; 9.08]	6.32	6.89	7.6	8.94	10.9
	Visit W5D1	Neopterin, µmol/l	13	8.45	[7.18; 9.74]	5.74	6.83	8.29	10.3	11.8
	Visit W9D1	Neopterin, µmol/l	13	8.35	[7.61; 9.16]	6.63	7.5	8.39	9.15	10.8
	Visit W13D1	Neopterin, µmol/l	13	8.14	[7.20; 9.08]	6.33	7.19	7.75	9.2	10
	End of study visit	Neopterin, µmol/l	13	8.24	[7.26; 9.25]	5.68	7.13	8.14	9.38	11.2

Neopterin, µmol/l - change

	Visit	Parameter	n	HL-estimate	95%-CI	Minimum	25%-Quantile	Median	75%-Quantile	Maximum
Total (N=13)	Visit W2D1	Neopterin, µmol/l	13	0.01	[-0.40; 0.58]	-0.97	-0.47	0.18	0.71	0.86
	Visit W5D1	Neopterin, µmol/l	13	0.325	[-0.43; 1.12]	-1.9	-0.4	0.37	0.98	5.3
	Visit W9D1	Neopterin, µmol/l	13	0.4	[-0.10; 0.87]	-0.81	-0.39	0.52	0.92	1.65
	Visit W13D1	Neopterin, µmol/l	13	0.095	[-0.61; 0.72]	-1.87	-0.65	-0.19	0.86	1.65
	End of study visit	Neopterin, µmol/l	13	0.175	[-0.53; 1.19]	-2.52	-0.28	0.08	0.44	3.04

3 CMR (per-protocol population)

LVEF, %

	Visit	Parameter	n	HL-estimate	95%-CI	Minimum	25%-Quantile	Median	75%-Quantile	Maximum
Total (N=13)	Baseline	LVEF, %	13	62.8	[60.8; 64.5]	57.5	61	63	65	66.5
	Visit W13D1	LVEF, %	13	62	[59.5; 64.8]	55	59	63	65	67
	End of study visit	LVEF, %	13	62	[59.5; 64.0]	56	60	63	64	68.4

LVEF, % - change

	Visit	Parameter	n	HL-estimate	95%-CI	Minimum	25%-Quantile	Median	75%-Quantile	Maximum
Total (N=13)	Visit W13D1	LVEF, %	13	-0.25	[-1.75; 1.00]	-10	-1	-0.39	0.5	2.5
	End of study visit	LVEF, %	13	-1.25	[-2.00; 0.25]	-3.5	-2	-1.5	-0.5	5.4

LVSVI, ml/m²

	Visit	Parameter	n	HL-estimate	95%-CI	Minimum	25%-Quantile	Median	75%-Quantile	Maximum
Total (N=13)	Baseline	LVSVI, ml/m²	13	48.3	[45.1; 52.4]	40.1	44.2	49.6	51.9	63.8
	Visit W13D1	LVSVI, ml/m²	13	46.2	[41.9; 51.0]	35.2	43.5	47.8	52.6	56.4
	End of study visit	LVSVI, ml/m²	13	45.8	[41.6; 49.6]	36.5	40.9	47	49.7	56.1

LVSVI, ml/m² - change

	Visit	Parameter	n	HL-estimate	95%-CI	Minimum	25%-Quantile	Median	75%-Quantile	Maximum
Total (N=13)	Visit W13D1	LVSVI, ml/m²	13	-0.85	[-3.54; 1.13]	-25.1	-3	-0.6	0.05	5.25
	End of study visit	LVSVI, ml/m²	13	-2.38	[-4.08; 0.12]	-21.1	-3.47	-3.2	-0.51	4.85

Cardiac index, l/min/m²

	Visit	Parameter	n	HL-estimate	95%-CI	Minimum	25%-Quantile	Median	75%-Quantile	Maximum
Total (N=13)	Baseline	Cardiac index, l/min/m²	13	2.85	[2.72; 3.05]	2.62	2.7	2.81	3	3.4
	Visit W13D1	Cardiac index, l/min/m²	13	2.72	[2.50; 2.96]	2.14	2.5	2.71	2.96	3.3
	End of study visit	Cardiac index, l/min/m²	13	2.75	[2.50; 2.95]	2.4	2.5	2.6	3.1	3.4

Cardiac index, l/min/m² - change

	Visit	Parameter	n	HL-estimate	95%-CI	Minimum	25%-Quantile	Median	75%-Quantile	Maximum
Total (N=13)	Visit W13D1	Cardiac index, l/min/m²	13	-0.15	[-0.32; 0.00]	-0.58	-0.25	-0.15	0	0.215
	End of study visit	Cardiac index, l/min/m²	13	-0.153	[-0.29; - 0.00]	-0.45	-0.29	-0.2	0	0.3

Mean global T1, ms

	Visit	Parameter	n	HL-estimate	95%-CI	Minimum	25%-Quantile	Median	75%-Quantile	Maximum
Total (N=13)	Baseline	Mean global T1, ms	13	1236	[1221; 1254]	1187	1220	1235	1254	1298
	Visit W13D1	Mean global T1, ms	13	1250	[1232; 1279]	1199	1241	1250	1279	1345
	End of study visit	Mean global T1, ms	13	1237	[1221; 1258]	1189	1211	1244	1257	1285

Mean global T1, ms - change

	Visit	Parameter	n	HL-estimate	95%-CI	Minimum	25%-Quantile	Median	75%-Quantile	Maximum
Total (N=13)	Visit W13D1	Mean global T1, ms	13	13.8	[-0.26; 35.8]	-20.8	-0.26	11.4	23.4	85
	End of study visit	Mean global T1, ms	13	3.3	[-13.6; 16.0]	-43.2	-9.5	6.4	16	30

Mean global T2, ms

	Visit	Parameter	n	HL-estimate	95%-CI	Minimum	25%-Quantile	Median	75%-Quantile	Maximum
Total (N=13)	Baseline	Mean global T2, ms	13	46.3	[45.53; 47.91]	44.3	45.0	46.8	47.4	51.5
	Visit W13D1	Mean global T2, ms	12	48.2	[46.59; 49.52]	44.6	46.6	48.2	49.5	51.8
	End of study visit	Mean global T2, ms	13	47.1	[45.27; 49.62]	43.0	45.3	46.7	49.3	53.9

Mean global T2, ms - change

	Visit	Parameter	n	HL-estimate	95%-CI	Minimum	25%-Quantile	Median	75%-Quantile	Maximum
Total (N=13)	Visit W13D1	Mean global T2, ms	12	1.37	[-0.43; .97]	-4.39	-0.41	1.52	2.77	6.59
	End of study visit	Mean global T2, ms	13	0.75	[-1.53; 3.15]	-4.46	-1.73	-0.03	3.23	7.07

global circumferential strain, %

	Visit	Parameter	n	HL-estimate	95%-CI	Minimum	25%-Quantile	Median	75%-Quantile	Maximum
Total (N=13)	Baseline	global circumferential strain, %	13	-17.7	[-18.8; -16.5]	-20.1	-19.3	-17.7	-16.8	-13.8
	Visit W13D1	global circumferential strain, %	13	-17.5	[-18.9; -16.3]	-22.5	-18.5	-16.8	-16.3	-15.6
	End of study visit	global circumferential strain, %	13	-17.4	[-18.9; -16.3	-20.8	-18.9	-17.4	-15.9	-14.2

global circumferential strain, % - change

	Visit	Parameter	n	HL-estimate	95%-CI	Minimum	25%-Quantile	Median	75%-Quantile	Maximum
Total (N=13)	Visit W13D1	global circumferential strain, %	13	-0.2	[-1.48; 1.57]	-5.78	-1.6	-0.2	1.63	3.65
	End of study visit	global circumferential strain, %	13	0.378	[-0.77; 1.28]	-5.17	-0.7	0.3	1.7	2.55

global longitudinal strain, %

	Visit	Parameter	n	HL-estimate	95%-CI	Minimum	25%-Quantile	Median	75%-Quantile	Maximum
Total (N=13)	Baseline	global longitudinal strain, %	13	-13.7	[-14.9; -12.4]	-17.4	-14	-13.7	-12.3	-10.8
	Visit W13D1	global longitudinal strain, %	13	-13.7	[-15.1; -12.6]	-16.8	-15	-13.7	-12.4	-10.4
	End of study visit	global longitudinal strain, %	13	-13.8	[-14.8; -12.6]	-16.4	-14.7	-13.2	-12.8	-10.5

global longitudinal strain, % - change

	Visit	Parameter	n	HL-estimate	95%-CI	Minimum	25%-Quantile	Median	75%-Quantile	Maximum

global longitudinal strain, % - change

	Visit	Parameter	n	HL-estimate	95%-CI	Minimum	25%-Quantile	Median	75%-Quantile	Maximum
Total (N=13)	Visit W13D1	global longitudinal strain, %	13	0.05	[-1.74; 1.15]	-4.24	-1.94	0.64	0.85	4.0
	End of study visit	global longitudinal strain, %	13	-0.003	[-1.25; 1.13]	-3.45	-1.26	0.45	1.04	3.52

4 Glucose metabolism (per-protocol population)

HbA1c, mmol/mol

	Visit	Parameter	n	HL-estimate	95%-CI	Minimum	25%-Quantile	Median	75%-Quantile	Maximum
Total (N=13)	Baseline	HbA1c, mmol/mol	13	38	[34.0; 41.0]	29	36	38	40	46
	Visit W13D1	HbA1c, mmol/mol	13	36	[33.5; 40.0]	31	33	36	39	49

HbA1c, mmol/mol - change

	Visit	Parameter	n	HL-estimate	95%-CI	Minimum	25%-Quantile	Median	75%-Quantile	Maximum
Total (N=13)	Visit W13D1	HbA1c, mmol/mol	13	-0.5	[-3.50; 1.50]	-7	-1	0	1	4

HOMA-insulin resistance, no unit

	Visit	Parameter	n	HL-estimate	95%-CI	Minimum	25%-Quantile	Median	75%-Quantile	Maximum
Total (N=13)	Baseline	HOMA-insulin resistance, no unit	13	2.15	[1.19; 2.96]	0.61	1.01	1.72	3.29	4.42
	Visit W13D1	HOMA-insulin resistance, no unit	13	1.89	[1.29; 2.94]	0.6	1.2	1.81	2.31	4.85

HOMA-insulin resistance, no unit - change

	Visit	Parameter	n	HL-estimate	95%-CI	Minimum	25%-Quantile	Median	75%-Quantile	Maximum
Total (N=13)	Visit W13D1	HOMA-insulin resistance, no unit	13	-0.01	[-0.35; 0.25]	-1.52	-0.35	0	0.23	1.05

5 Bile acids (per-protocol population)

Trough - Total Bile Acids, nmol/L

	Visit	Parameter	n	Mean	Standard deviation	Minimum	25%-Quantile	Median	75%-Quantile	Maximum
Total (N=13)	Visit W1D1 1b	Trough - Total Bile Acids, nmol/L	13	2026	2158	404	760	983	1603	7308
	Visit W13D1	Trough - Total Bile Acids, nmol/L	13	9922	7357	2685	5195	6371	17400	23957
	End of study visit	Trough - Total Bile Acids, nmol/L	13	1928	1666	369	909	1349	2522	6140

AUC/ Cmax - GUDCA, nmol/L

	Visit	Parameter	n	Mean	Standard deviation	Minimum	25%-Quantile	Median	75%-Quantile	Maximum
Total (N=13)	Visit W1D1 1b	AUC - GUDCA	13	1325	955	201	514	1041	1998	3488
		Cmax - GUDCA	13	487	337	59.9	159	461	649	1137
	Visit W13D1	AUC - GUDCA	13	6280	2358	2429	5181	6229	7151	10666
		Cmax - GUDCA	13	2079	930	699	1665	1924	2344	4417

Trough - GUDCA, nmol/L

	Visit	Parameter	n	Mean	Standard deviation	Minimum	25%-Quantile	Median	75%-Quantile	Maximum
Total (N=13)	Visit W1D1 1b	Trough - GUDCA, nmol/L	13	63.9	109	10.8	16.2	26.8	39	408
	Visit W13D1	Trough - GUDCA, nmol/L	13	292	251	39.5	97.9	203	482	733
	End of study visit	Trough - GUDCA, nmol/L	13	67	77.7	12.4	26.4	35.4	46.5	240

AUC/ Cmax - GCA, nmol/L

	Visit	Parameter	n	Mean	Standard deviation	Minimum	25%-Quantile	Median	75%-Quantile	Maximum
Total (N=13)	Visit W1D1 1b	AUC - GCA	13	10450	9322	2311	4208	7000	10674	31786
		Cmax - GCA	13	4197.1	3930	936	1505	2926	3858	12688
	Visit W13D1	AUC - GCA	13	91693	51612	28049	63864	69417	109865	212962
		Cmax - GCA	13	33819	21012	8363	21004	23784	42756	79462

Trough - GCA, nmol/L

	Visit	Parameter	n	Mean	Standard deviation	Minimum	25%-Quantile	Median	75%-Quantile	Maximum
Total (N=13)	Visit W1D1 1b	Trough - GCA, nmol/L	13	141	141	36.7	56.6	69.9	163	522
	Visit W13D1	Trough - GCA, nmol/L	13	1500	1194	136	817	1129	2006	3745

Trough - GCA, nmol/L

	Visit	Parameter	n	Mean	Standard deviation	Minimum	25%-Quantile	Median	75%-Quantile	Maximum
	End of study visit	Trough - GCA, nmol/L	13	141	118	48.3	81	106	121	478

AUC/ Cmax - TUDCA, nmol/L

	Visit	Parameter	n	Mean	Standard deviation	Minimum	25%-Quantile	Median	75%-Quantile	Maximum
Total (N=13)	Visit W1D1 1b	AUC - TUDCA	13	158	129	31.5	61.3	141	200	426
		Cmax - TUDCA	13	57.9	53.9	7.3	19.54	47.9	68	180
	Visit W13D1	AUC - TUDCA	13	1107	739	140	520	1036	1484	2741
		Cmax - TUDCA	13	407	287	44	239	329	577	892

Trough - TUDCA, nmol/L

	Visit	Parameter	n	Mean	Standard deviation	Minimum	25%-Quantile	Median	75%-Quantile	Maximum

Trough - TUDCA, nmol/L

	Visit	Parameter	n	Mean	Standard deviation	Minimum	25%-Quantile	Median	75%-Quantile	Maximum
Total (N=13)	Visit W1D1 1b	Trough - TUDCA, nmol/L	13	6.25	3.84	1.75	4.04	5.81	7.4	15.7
	Visit W13D1	Trough - TUDCA, nmol/L	13	18.3	9.85	5.18	10.8	19.2	26.2	35.4
	End of study visit	Trough - TUDCA, nmol/L	13	7.13	5.81	3.11	4.47	5.77	6.16	25.1

AUC/ Cmax - TCA, nmol/L

	Visit	Parameter	n	Mean	Standard deviation	Minimum	25%-Quantile	Median	75%-Quantile	Maximum
Total (N=13)	Visit W1D1 1b	AUC - TCA	13	2029	2064	327	650	1371	2717	7575
		Cmax - TCA	13	906	976	118	254	638	1163	3462
	Visit W13D1	AUC - TCA	13	30416	23921	2055	12483	20876	36155	74419
		Cmax - TCA	13	11315	9049	681	4109	7352	16975	26932

Trough - TCA, nmol/L

	Visit	Parameter	n	Mean	Standard deviation	Minimum	25%-Quantile	Median	75%-Quantile	Maximum
Total (N=13)	Visit W1D1 1b	Trough - TCA, nmol/L	13	20.5	15.2	7.77	10.2	15.2	24.0	59.5
	Visit W13D1	Trough - TCA, nmol/L	13	228	203	18.4	103	127	276	748
	End of study visit	Trough - TCA, nmol/L	13	25.7	21.4	8.21	15.5	18.3	22.7	87.5

AUC/ Cmax - CA, nmol/L

	Visit	Parameter	n	Mean	Standard deviation	Minimum	25%-Quantile	Median	75%-Quantile	Maximum
Total (N=13)	Visit W1D1 1b	AUC - CA	13	994	1909	0	210	345	825	7174
		Cmax - CA	13	590	1036	0	91	145	273	3329
	Visit W13D1	AUC - CA	13	3159	5470	374	727	1159	2290	20210
		Cmax - CA	13	1436	2022	85.6	245	301	1420	5190

Trough - CA, nmol/L

	Visit	Parameter	n	Mean	Standard deviation	Minimum	25%-Quantile	Median	75%-Quantile	Maximum
Total (N=13)	Visit W1D1 1b	Trough - CA, nmol/L	13	341	902	0	25.4	80	208	3329
	Visit W13D1	Trough - CA, nmol/L	13	893	1699	58.8	64.4	137	321	5190
	End of study visit	Trough - CA, nmol/L	13	270	611	0	0	16.2	49.4	1928

AUC/ Cmax - UDCA, nmol/L

	Visit	Parameter	n	Mean	Standard deviation	Minimum	25%-Quantile	Median	75%-Quantile	Maximum
Total (N=13)	Visit W1D1 1b	AUC - UDCA	13	351	207	108	242	255	522	725
		Cmax - UDCA	13	109	58.7	43.7	69.4	84.1	132	223
	Visit W13D1	AUC - UDCA	13	583	457	173	304	458	553	1672
		Cmax - UDCA	13	159	99.3	56.4	91.1	109	228	354

Trough - UDCA, nmol/L

	Visit	Parameter	n	Mean	Standard deviation	Minimum	25%-Quantile	Median	75%-Quantile	Maximum
Total (N=13)	Visit W1D1 1b	Trough - UDCA, nmol/L	13	69.5	55.2	6.8	43.7	55	84.1	223
	Visit W13D1	Trough - UDCA, nmol/L	13	90.2	99.2	21	34.4	53.0	77.7	354
	End of study visit	Trough - UDCA, nmol/L	13	50.9	37.5	8.08	35.7	41.2	51.1	164

AUC/ Cmax - GCDCA, nmol/L

	Visit	Parameter	n	Mean	Standard deviation	Minimum	25%-Quantile	Median	75%-Quantile	Maximum
Total (N=13)	Visit W1D1 1b	AUC - GCDCA	13	23510	15822	9142	10407	18842	31997	60922
		Cmax - GCDCA	13	9241	7101	2994	4524	7032	10678	27843
	Visit W13D1	AUC - GCDCA	13	135421	57545	52724	100786	132099	161671	252352
		Cmax - GCDCA	13	45681	20823	13218	34610	41877	60955	87814

Trough - GCDCA, nmol/L

	Visit	Parameter	n	Mean	Standard deviation	Minimum	25%-Quantile	Median	75%-Quantile	Maximum
Total (N=13)	Visit W1D1 1b	Trough - GCDCA, nmol/L	13	537	657	68.4	151	322	526	2090
	Visit W13D1	Trough - GCDCA, nmol/L	13	3559	2745	317	1521	2691	5802	7867
	End of study visit	Trough - GCDCA, nmol/L	13	568	682	108	226	353	489	2546

AUC/ Cmax - GDCA, nmol/L

	Visit	Parameter	n	Mean	Standard deviation	Minimum	25%-Quantile	Median	75%-Quantile	Maximum
Total (N=13)	Visit W1D1 1b	AUC - GDCA	13	8421	5170	3329	4604	6543	11965	17622
		Cmax - GDCA	13	3271	2038	1245	1652	2661	5014	7196
	Visit W13D1	AUC - GDCA	13	75502	45713	10883	34602	82736	101006	156822
		Cmax - GDCA	13	26639	18984	2907	13997	25864	30030	74754

Trough - GDCA, nmol/L

	Visit	Parameter	n	Mean	Standard deviation	Minimum	25%-Quantile	Median	75%-Quantile	Maximum
Total (N=13)	Visit W1D1 1b	Trough - GDCA, nmol/L	13	176	224	46	49.4	69	215	847
	Visit W13D1	Trough - GDCA, nmol/L	13	1596	1390	155	621	1317	2181	4442
	End of study visit	Trough - GDCA, nmol/L	13	254	404	35.7	77.8	118.6	201	1557

AUC/ Cmax - TCDCA, nmol/L

	Visit	Parameter	n	Mean	Standard deviation	Minimum	25%-Quantile	Median	75%-Quantile	Maximum
Total (N=13)	Visit W1D1 1b	AUC - TCDCA	13	4750	4107	541	1851	3764	6587	14596
		Cmax - TCDCA	13	1917	1710	202	750	1354	2464	5935
	Visit W13D1	AUC - TCDCA	13	38188	30700	4256	16786	31600	46793	105955
		Cmax - TCDCA	13	13883	11722	1379	5959	9077	17505	41083

Trough - TCDCA, nmol/L

	Visit	Parameter	n	Mean	Standard deviation	Minimum	25%-Quantile	Median	75%-Quantile	Maximum
Total (N=13)	Visit W1D1 1b	Trough - TCDCA, nmol/L	13	61.9	69.1	14	27.1	35.6	52.0	237
	Visit W13D1	Trough - TCDCA, nmol/L	13	448	301	23.1	262	420	554	1002
	End of study visit	Trough - TCDCA, nmol/L	13	71.4	98.8	15	36	37.6	51.0	391

AUC/ Cmax - TDCA, nmol/L

	Visit	Parameter	n	Mean	Standard deviation	Minimum	25%-Quantile	Median	75%-Quantile	Maximum
Total (N=13)	Visit W1D1 1b	AUC - TDCA	13	1926.3	1497.3	213.49625	703.10125	1076.4	3370.555	4262.9113
		Cmax - TDCA	13	786.98	638.38	97.99	274.6	451.54	1353.98	1991.57
	Visit W13D1	AUC - TDCA	13	21894	14356	905.98875	11535.473	22286	30385.218	45862.355
		Cmax - TDCA	13	7898.6	6029.9	248.18	3832.27	7170.7	9523.9	21289.41

Trough - TDCA, nmol/L

	Visit	Parameter	n	Mean	Standard deviation	Minimum	25%-Quantile	Median	75%-Quantile	Maximum
Total (N=13)	Visit W1D1 1b	Trough - TDCA, nmol/L	13	24.6	34.5	4.0	7.34	13.7	24.1	136
	Visit W13D1	Trough - TDCA, nmol/L	13	248	246	12	76.3	104	305	832
	End of study visit	Trough - TDCA, nmol/L	13	47.4	104	6.87	10.3	16.6	22.3	390

AUC/ Cmax - CDCA, nmol/L

	Visit	Parameter	n	Mean	Standard deviation	Minimum	25%-Quantile	Median	75%-Quantile	Maximum
Total (N=13)	Visit W1D1 1b	AUC - CDCA	13	1827	1869	331	504	1083	2677	6603
		Cmax - CDCA	13	903	817	113	300	511	1580	2483
	Visit W13D1	AUC - CDCA	13	3058	3108	408	674	1816	4539	11375
		Cmax - CDCA	13	1311	1207	109	184	1024	2273	3597

Trough - CDCA, nmol/L

	Visit	Parameter	n	Mean	Standard deviation	Minimum	25%-Quantile	Median	75%-Quantile	Maximum
Total (N=13)	Visit W1D1 1b	Trough - CDCA, nmol/L	13	373	686	20.4	60.5	112.9	234	2483
	Visit W13D1	Trough - CDCA, nmol/L	13	623	1206	59.1	86.8	146.1	175	3597
	End of study visit	Trough - CDCA, nmol/L	13	177	266	13.1	34.8	70.2	131	875

AUC/ Cmax - DCA, nmol/L

	Visit	Parameter	n	Mean	Standard deviation	Minimum	25%-Quantile	Median	75%-Quantile	Maximum
Total (N=13)	Visit W1D1 1b	AUC - DCA	13	194	1742	514	683	1328	3304	5266
		Cmax - DCA	13	676	638	118	193	377	989	1805
	Visit W13D1	AUC - DCA	13	3882	2672	656	1522	3589	5849	9443
		Cmax - DCA	13	1090	714	176	498	860	1413	2395

AUC/ Cmax - DCA, nmol/L

	Visit	Parameter	n	Mean	Standard deviation	Minimum	25%-Quantile	Median	75%-Quantile	Maximum

Trough - DCA, nmol/L

	Visit	Parameter	n	Mean	Standard deviation	Minimum	25%-Quantile	Median	75%-Quantile	Maximum
Total (N=13)	Visit W1D1 1b	Trough - DCA, nmol/L	13	177	94.4	59.5	117	160	260	347
	Visit W13D1	Trough - DCA, nmol/L	13	365	273	85.7	91.1	319	596	842
	End of study visit	Trough - DCA, nmol/L	13	210	246	24.2	82.1	119	210	775

AUC/ Cmax - GLCA, nmol/L

	Visit	Parameter	n	Mean	Standard deviation	Minimum	25%-Quantile	Median	75%-Quantile	Maximum
Total (N=13)	Visit W1D1 1b	AUC - GLCA	13	546	435	123	221	437	733	1738
		Cmax - GLCA	13	203	178	33.5	65.3	141	239	606

AUC/ Cmax - GLCA, nmol/L

	Visit	Parameter	n	Mean	Standard deviation	Minimum	25%-Quantile	Median	75%-Quantile	Maximum
	Visit W13D1	AUC - GLCA	13	2388	1006	987	1747	2326	2757	4552
		Cmax - GLCA	13	887	419	272	615	880	1075	1925

Trough - GLCA, nmol/L

	Visit	Parameter	n	Mean	Standard deviation	Minimum	25%-Quantile	Median	75%-Quantile	Maximum
Total (N=13)	Visit W1D1 1b	Trough - GLCA, nmol/L	13	10.4	6.52	6.68	7.69	8.51	9.48	31.5
	Visit W13D1	Trough - GLCA, nmol/L	13	31	33.8	7.54	17.7	20.5	24.9	139
	End of study visit	Trough - GLCA, nmol/L	13	11.7	9.71	5.66	7.14	8.56	11.2	42.7

AUC/ Cmax - TLCA, nmol/L

	Visit	Parameter	n	Mean	Standard deviation	Minimum	25%-Quantile	Median	75%-Quantile	Maximum
Total (N=13)	Visit W1D1 1b	AUC - TLCA	13	106	82.3	38.5	50.4	65.6	142	320
		Cmax - TLCA	13	34.9	31.8	9.47	15.9	19.2	41.1	120
	Visit W13D1	AUC - TLCA	13	583	441	72.9	200	565	803	1709
		Cmax - TLCA	13	215	182	15.7	101	196	251	698

Trough - TLCA, nmol/L

	Visit	Parameter	n	Mean	Standard deviation	Minimum	25%-Quantile	Median	75%-Quantile	Maximum
Total (N=13)	Visit W1D1 1b	Trough - TLCA, nmol/L	13	4.46	2.49	0	3.67	4.30	4.64	11.5
	Visit W13D1	Trough - TLCA, nmol/L	13	9.01	5.82	4.35	5.78	6.44	10.0	23.9
	End of study visit	Trough - TLCA, nmol/L	13	5.16	3.61	2.55	3.87	4.42	4.7	16.9

AUC/ Cmax - LCA, nmol/L

	Visit	Parameter	n	Mean	Standard deviation	Minimum	25%-Quantile	Median	75%-Quantile	Maximum
Total (N=13)	Visit W1D1 1b	AUC - LCA	13	120	11.3	101	113	121	124	140
		Cmax - LCA	13	24.7	6.29	18.4	20.8	24.2	25.8	43.7
	Visit W13D1	AUC - LCA	13	135	25.5	99.3	120	133	160	173
		Cmax - LCA	13	27.2	7.3	18.1	21.5	26.8	31.2	40.7

Trough - LCA, nmol/L

	Visit	Parameter	n	Mean	Standard deviation	Minimum	25%-Quantile	Median	75%-Quantile	Maximum
Total (N=13)	Visit W1D1 1b	Trough - LCA, nmol/L	13	19.7	2.65	16.3	18.1	18.7	21.8	24.5
	Visit W13D1	Trough - LCA, nmol/L	13	20.5	5.31	14.8	17.2	18.4	23.6	32.8
	End of study visit	Trough - LCA, nmol/L	13	22.7	8.81	13.9	17.7	19.7	24.6	48.2

6 Safety analysis: ECG conduction intervals (safety population)

	Screening (n = 14)	W13D1 (n = 13)
**Heart rate**	57.93 ± 7.45	60.08 ± 8.22
**PR (ms)**	173.50 ± 26.48	169.31 ± 23.74
**QRS (ms)**	96.93 ± 11.22	97.77 ± 10.24
**QTcF (ms)**	410.29 ± 16.24	419.15 ± 16.10
